# African Herbal Remedies with Antioxidant Activity: A Potential Resource Base for Wound Treatment

**DOI:** 10.1155/2018/4089541

**Published:** 2018-11-22

**Authors:** Mary Gulumian, Ewura Seidu Yahaya, Vanessa Steenkamp

**Affiliations:** ^1^National Institute for Occupational Health, Johannesburg, South Africa; ^2^Haematology and Molecular Medicine, University of the Witwatersrand, Johannesburg, South Africa; ^3^Department of Pharmacology, Faculty of Health Sciences, University of Pretoria, Pretoria, South Africa; ^4^Department of Pharmacology, University of Cape Coast, Cape Coast, Ghana

## Abstract

The use of traditional herbal remedies as alternative medicine plays an important role in Africa since it forms part of primary health care for treatment of various medical conditions, including wounds. Although physiological levels of free radicals are essential to the healing process, they are known to partly contribute to wound chronicity when in excess. Consequently, antioxidant therapy has been shown to facilitate healing of such wounds. Also, a growing body of evidence suggests that, at least, part of the therapeutic value of herbals may be explained by their antioxidant activity. This paper reviews African herbal remedies with antioxidant activity with the aim of indicating potential resources for wound treatment. Firstly, herbals with identified antioxidant compounds and, secondly, herbals with proven antioxidant activity, but where the compound(s) responsible for the activity has not yet been identified, are listed. In the latter case it has been attempted to ascribe the activity to a compound known to be present in the plant family and/or species, where related activity has previously been documented for another genus of the species. Also, the tests employed to assess antioxidant activity and the potential caveats thereof during assessment are briefly commented on.

## 1. Introduction

Human cells are continuously exposed to exogenous oxidants as well as to those produced endogenously during normal physiological processes. Antioxidants form part of protective mechanisms that exist in human cells to scavenge and neutralize these oxidants. Oxidants such as the reactive oxygen species (ROS) and reactive nitrogen species (RNS) are involved in several diseases [1, 2]. Antioxidant defenses are defective in these diseases and therefore it is possible to limit oxidative damage and ameliorate disease progression with antioxidant supplementation [3].

**Figure 1:**
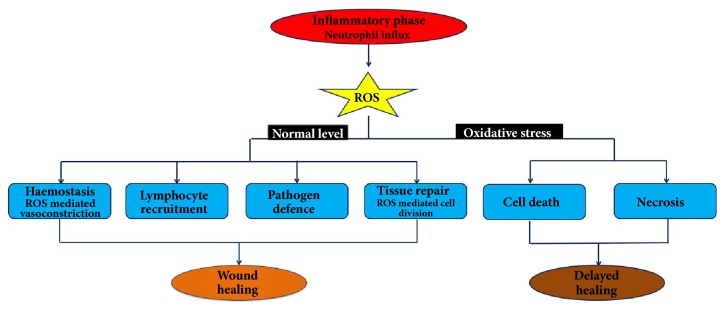
Role of reactive oxygen species (ROS) in the wound healing process.

With reference to wounds, antioxidants play pivotal roles that consequently restore normalcy to injured skin. Basal levels of ROS and other free radicals are essential in almost all phases of the wound healing process (Figure 1) [4]. During haemostasis, ROS regulates the constriction of blood vessels to limit loss of blood. Furthermore, ROS facilitates the migration of neutrophils and monocytes from surrounding blood vessels towards the injury site. The presence of ROS and other free radicals in the wound vicinity during the inflammatory phase of the healing process is also required for infection control and general maintenance of sterility. Finally, ROS promotes the proliferation of keratinocytes, endothelial cells, and fibroblasts, thereby enhancing angiogenesis and collagen deposition. However, uncontrolled release of ROS could cause oxidative stress, resulting in cellular and tissue damage, thereby causing delayed healing [1].

To keep ROS within physiological levels, antioxidants serve as electron donors, thereby preventing them from capturing electrons from other molecules which ultimately leads to their destruction [4]. Both nonenzymatic antioxidants such as glutathione, ascorbic acid, and *α*-tocopherol, as well as enzymatic antioxidants like catalase and peroxiredoxin, have shown potential to normalize high ROS levels and thus stimulate healing [4]. By normalizing ROS, antioxidants can enhance their physiological roles and thereby accelerate the wound healing process. Naturally occurring antioxidants are generally favoured over their synthetic counterparts, as the latter are suspected to cause or promote negative health effects [5]. This has resulted in the restricted use of synthetic antioxidants in several countries [6].

This review provides a comprehensive list of African medicinal plants and isolated compounds with antioxidant activities, with the aim of highlighting the continent's rich herbal resource base for possible management of wounds and allied conditions. Previous reviews have listed a number of these African medicinal plants with antioxidant properties [7–9]. The present work has therefore aimed to expand the list to include medicinal plant species with antioxidant properties that are used in different African countries including those from Madagascar and Mauritius. For the sake of inclusivity, plants that have been shown to contain compounds that hold the potential of being novel antioxidants are also considered. In addition, those with anti-inflammatory properties were also included due to an earlier observation that the anti-inflammatory activities of the same extracts could be explained, at least in part, by their antioxidant properties [10]. Additional efforts were also made to include information, where available, on their vernacular names, their regional distribution, and medicinal use and plant parts used for these preparations or for the isolation of the antioxidant ingredient(s).  Table 1 lists medicinal plants that have been investigated and have confirmed antioxidant and/or anti-inflammatory activity and that contain compounds which are known to have such activities.  Table 2 on the other hand lists medicinal plants that have confirmed antioxidant activity but the compounds responsible for their antioxidant property have not yet been identified.

Many edible and culinary herbs and condiments were also included in these two tables as they were used in certain instances as medicinal herbs to treat diseases. These included fruits and seeds of* Balanites aegyptiaca*, leaves of* Boscia senegalensis*, leaves of* Entada africana* and seeds of* Parkia biglobosa*, from Niger [11], also leaves, seeds, and stem-bark of* Mangifera indica* from Benin and Burkina Faso [12, 13], leaves of* Cynara scolymus* from Ethiopia [14, 15], leaves of* Aspalathus linearis* from South Africa [16–21], leaves of* Cinnamomum zeylanicum* from Madagascar and Ethiopia [22–24], essential oils from the bark and leaves of* Ravensara aromatica* from Madagascar [23, 25], buds of* Syzygium aromaticum* from Madagascar [23], seeds of* Trigonella foenumgraecum* from Ethiopia and Morocco [26–28], and oils in seeds of* Nigella sativa* from African countries of the Mediterranean region [29–31].

## 2. Tests Used to Assess Antioxidant Activities of African Medicinal Plant Extracts

**Figure 2:**
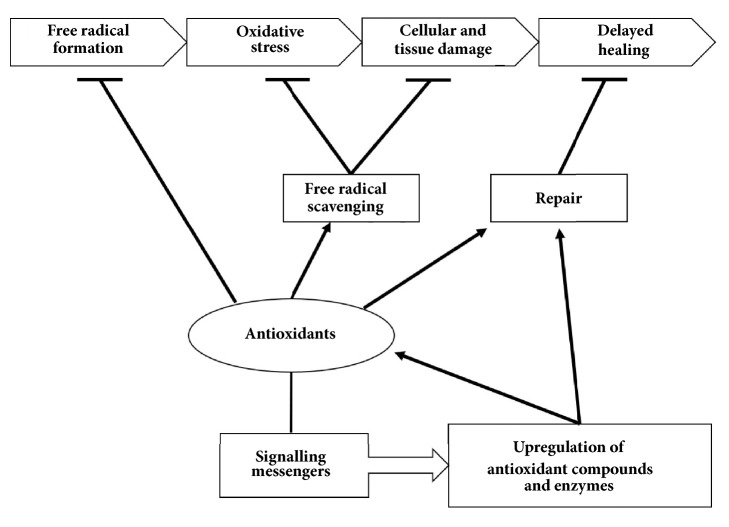
Mechanism of antioxidant action in wounds.

A variety of test systems were employed to assess the antioxidant properties of the medicinal plant extracts and compounds listed in Tables 1 and 2. A comprehensive list of the methods used in antioxidant activity determination, as well as their merits and demerits, has already been published [343–346]. The methods used in the determination of antioxidant activity of natural products and isolated compounds result in varied outcomes when the same samples are tested in different laboratories and by other researchers [347]. Furthermore, results of different methods cannot be correlated, as contradictory results are usually obtained. Hence, although several assays are available, none of them is capable of accurately and completely determining the antioxidant activity of a test substance because of the complex nature of the redox-antioxidant system* in vivo* (Figure 2). Based on this complexity, antioxidants are broadly classified as (i) inhibitors of free radical formation, (ii) free radical scavengers, (iii) cellular and tissue damage repairers, and (iv) signalling messengers [347].

The inhibition of free radical formation could protect against oxidative damage by suppressing the formation of active ROS/RNS. This typically involves reduction or inhibition of substrates required for free radical formation such as metal ions like iron (Fe) and copper (Cu). The sequestration of these metal ions by antioxidant compounds like ellagic acid and glutathione is known to suppress formation of hydrogen peroxide (H_2_O_2_) and other free radicals [348, 349]. Furthermore, increasing evidence suggests a relationship between metal overload and several chronic diseases through the induction of oxidative stress [350]. Therefore, inhibition of free radical formation using metal ions as targets could be useful therapeutically. Antioxidant assays designed for this purpose include the cupric and ferric reducing antioxidant power (CUPRAC/FRAP). These methods measure the ability of antioxidants to reduce cupric (Cu^2+^) and ferric (Fe^3+^) ions, respectively.

Another mechanism by which antioxidants act is through the suppression of oxidative stress by directly scavenging active free radicals. Most commonly reported antioxidant assays such as 2,2′-azino-bis(3-ethylbenzthiazoline-6-sulfonic acid) (ABTS), 2,2′-diphenyl-p-picrylhydrazyl radical (DPPH), oxygen radical absorbance capacity (ORAC), Trolox equivalent antioxidant capacity (TEAC), total oxyradical scavenging capacity (TOSC), and total radical antioxidant parameter (TRAP) are focused on testing the ability to scavenge free radicals. Furthermore, there are diverse cellular antioxidant assays that assess the ability of antioxidant compounds and substances to protect cells against excessive free radical generation. Such assays involve the use of a fluorescent compound such as 2,7-dichlorofluoroscein to determine the ability of test samples to quench intracellularly generated free radicals and inhibit radical formation and lipid peroxidation [345].

There are also numerous reports of the ability of antioxidants to repair damaged tissues and improve healing. Topical application of kojic acid and deferiprone, two compounds with the ability to scavenge free radicals, enhanced healing of wounds in rats [351]. Also, the mitochondria-targeted antioxidant, 10-(6′-plastoquinonyl) decyltriphenylphosphonium, accelerated wound closure, stimulated epithelialization, granulation tissue formation, and vascularization, and lowered lipid peroxidation in mice [352]. Moreover, an antioxidant peptide (cathelicidin-OA1) promoted wound healing in a mouse model with full-thickness skin wounds, accelerated reepithelialization and granulation tissue formation by enhancing the recruitment of macrophages to the wound site, and induced cell proliferation and migration [353]. Some antioxidants have also been reported to contribute to healing by enhancing the activity of endogenous antioxidant compounds and enzymes. The induction of the nuclear factor E2-related factor 2-(Nrf2) mediated antioxidative pathway by a rhomboid family protein (RHBDF2) promoted healing of injured tissues, suggesting a relationship between antioxidant gene induction and healing [354]. Niconyl-peptide enhanced wound healing and protected against hydrogen peroxide-induced cell death by increasing the expression of Nrf2 expression in human keratinocytes [355].

The most common tests used to determine the antioxidant activity of samples included the assessment of the ability to scavenge free radicals such as DPPH, ABTS^+^ [16, 19, 35, 62, 85, 94, 98, 99, 139, 158, 175, 184, 187, 266, 282, 302, 356–364], or the hydroxyl radicals [79, 188, 267, 365, 366], as well as the hydroperoxyl radicals by the Briggs-Rauscher reaction [104]. The ability of the extracts to chelate metal ions was also determined as further indication of their ability to contribute in the reduction of free radicals such as the hydroxyl radical [114]. In addition, assessment of the ability of these medicinal plant extracts to protect against lipid peroxidation was also included, which in turn was measured by the malondialdehyde-thiobarbituric acid (MDA) test [320, 367], the modified thiobarbituric acid reactive species (TBARS) assay [18, 22], or conjugated diene formation [367]. Moreover, lipid peroxidation was assessed using the fluorescent probe, diphenyl-1-pyrenylphosphine (DPPP) [188], or using the inhibition of Cu(2+)-mediated oxidation of human low-density lipoprotein (LDL) [187, 367]. The ability of extracts to protect against damage to DNA using the Comet assay was also employed [114, 188].

The antioxidant capacity of the medicinal plant extracts was determined using either the TEAC or FRAP assays [11, 85, 302, 313, 321, 368]. The ability of extracts to modulate the gene expression of the antioxidant enzymes, such as Cu, Zn-superoxide dismutase (Cu, Zn-SOD), Mn-superoxide dismutase (Mn-SOD), catalase, and glutathione peroxidase (GPx), was also used as a measure of their antioxidant properties [293]. The photochemilumiescence (PLC) assay is a more recent antioxidant capacity assessment method and was employed for the evaluation of antioxidant capacity of baobab fruit pulp extracts [369].

Anti-inflammatory properties of these extracts were assessed by their ability to inhibit 5-lipoxygenases [94, 370, 371] or cyclooxygenase (COX-1 and COX-2) activities [65, 275, 317, 372, 373]. Using the former [374] and the latter [264, 331] methodologies, respectively, a great number of South African medicinal plant extracts were screened for their anti-inflammatory properties. The effect of medicinal extracts on the biosynthesis of different prostaglandins was assessed as a measure of their anti-inflammatory effect [239, 337, 375]. Extracts of* Podocarpus* species were shown to inhibit the activities of the COX enzymes [317]. Once again, using this test, the anti-inflammatory properties of the aqueous and ethanolic extracts of 39 plants used in traditional Zulu medicine were screened [376]. The Hen's Egg Test-Chorioallantoic Membrane (HET-CAM) assay which utilizes the CAM's capillary system in bred hen eggs was also used to assess the anti-inflammatory activity through antiangiogenic effects of the ethanol and aqueous extracts of* Drosera rotundifolia* and* D. madagascariensis* [155].

The antioxidant and anti-inflammatory abilities of the herbal extracts were further assessed by evaluating their ability to control the production of ROS produced by oxidative burst in neutrophils stimulated with L-formyl-L-methionyl-L-leucyl-L-phenylalanine (FMLP) [21, 246]. The inhibition of neutrophils elastase was used as a measure of anti-inflammatory property and it was proposed that the presence of flavonoids such as hyperoside, quercetin, and isoquercitrin in* D. rotundifolia* [377] and five flavonoid compounds in two* Polypodium* species (*P. decumanum* and* P. triseriale*) [378] were thought to contribute to this anti-inflammatory activity. These and other* in vitro* tests were used to assess the antioxidant properties of three Ghanaian species:* Spathodea campanulata*,* Commelina diffusa*, and* Secamone afzelii* [63].

Inflammation is a complex mechanism with many pathways. Several extracts derived from medicinal plants have been shown to modulate or inhibit the activities of mediators of inflammation. For instance, kolaviron, a bioflavonoid compound isolated from the seeds of* Garcinia kola*, has been reported to possess anti-inflammatory and antioxidant activities via its effects on COX-2 and inducible nitric oxide synthase (iNOS) by inhibiting the expression of nuclear factor kappa B (NF-*κ*B) [115]. Quercetin is a flavonoid molecule ubiquitous in nature and functions as an antioxidant and anti-inflammatory agent. Dose- and time-dependent effects of quercetin have been investigated on proinflammatory cytokine expression and iNOS, focusing on its effects on NF-*κ*B signal transduction pathways in lipopolysaccharide-stimulated RAW 264.7 cells by using real time polymerase chain reaction (RT-PCR) and immunoblotting. Curcumin, a yellow pigment of turmeric, has been shown to exhibit anti-inflammatory activity. Curcumin has been found effective in the treatment or control of chronic inflammatory conditions such as rheumatism, atherosclerosis, type II diabetes, and cancer [203]. Calixto et al. reported that the anti-inflammatory action of active spice-derived components results from the disruption of the production of various inflammatory proteins (e.g., cytokines such as tumour necrosis factor-alpha (TNF-*α*), iNOS, and COX-2) [379].

Animal studies were also conducted to assess the antioxidant properties of several medicinal extracts. The antioxidant potential of* Hypericum perforatum,* containing many polyphenolic compounds, was evaluated on splanchnic artery occlusion (SAO) shock-mediated injury [477] and also against elevated brain oxidative status induced by amnestic dose of scopolamine in rats [126]. Some medicinal plant extracts were tested for their ability to protect against carbon tetrachloride-, 2-acetylaminofluorene- (2-AAF-), and galactosamine-induced liver as well as aflatoxin B1-(AFB1-)induced genotoxicity. Using this test, it was found that an extract of* Garcinia kola* seeds [116, 478, 479], a decoction of* Trichilia roka* root [270], extracts of* Entada africana* [442], and* Thonningia sanguinea* [98, 480] possessed protective abilities. The antioxidant properties of plant extracts against potassium bromate (KBrO(3))-induced kidney damage showed the ability of* G. kola* seed extract to protect the kidneys [481].

Animal studies were also used to assess the anti-inflammatory ability of a great number of medicinal plant extracts using the carrageenan-induced rat paw oedema model. Plants investigated include seed extracts of* Picralima nitida* [399], crude methanol extract of the root of* Moringa oleifera* [469], powdered leaves and root of* Mallotus oppositifolium* [167], methanolic extract of* Picralima nitida* fruit [400], hot water extract of* Alstonia boonei* root-bark,* Rauvolfia vomitoria* root-bark, and* Elaeis guineensis* nuts [56], secondary root aqueous extract of* Harpagophytum procumbens* [303], crude extracts of* Sphenocentrum jollyanum* [272], aqueous and methanolic extracts of* Hypoxis hemerocallidea* corm [482], aqueous and methanolic extracts of* Sclerocarya birrea* stem-bark [483], aqueous extract of* Mangifera indica* stem-bark [13], aqueous extracts of* Leonotis leonurus* leaves [484], leaf extracts of* Bryophyllum pinnatum* [148], methanol extracts of the stem-bark of* Alstonia boonei* [485], aerial parts of* Amaranthus caudatus *[486], methanolic extracts of* Kigelia pinnata* flower [415], and leaf and twig extracts of* Dorstenia barteri* [276]. In all of these studies, the anti-inflammatory effect against carrageenan-induced rat paw oedema was attributed to flavonoids and other polyphenolic compounds. Animal tests also employed to assess the anti-inflammatory effects of the medicinal plant extracts included inflammatory cell response such as neutrophil chemotaxis and degranulation [112, 487], antiatherosclerosis effects [486], and pain assessment in experimental animals [117].

The effect of the medicinal plants on the induction or inhibition of drug metabolizing enzymes was also studied in animals. The effect of the aqueous extract of* Thonningia sanguinea* on 7-ethoxyresorufin O-deethylase (EROD, CYP1A1), 7-pentoxyresorufin O-dealkylase (PROD, CYP2B1/2), 7-methoxyresorufin O-demethylase (MROD, CYP1A2), aniline hydroxylase (aniline, CYP2E1),* p*-nitrophenol hydroxylase (PNPH, CYP2E1), and erythromycin N-demethylase (ERDM, CYP3A1) in rat liver was found to selectively modulate CYP isoenzymes [100] and suppress CYP3A2 and CYP1A2 gene expression [101].

## 3. Compounds Isolated from African Medicinal Plant Extracts with Confirmed Antioxidant Activities

Several medicinal plant extracts were studied at research centres in African countries for their antioxidant properties. The major findings of these investigations have indicated that, in addition to known antioxidant compounds such as ascorbic acid in the seeds of* Parkia biglobosa* [204] and fruits pulp of* Adansonia digitata* [369], alpha-tocopherol in methanol extracts of the stems of* Secamone afzelii* [62] or from the seeds [38] and methanol extracts of leaves of* Amaranthus caudatus* [39], and apigenin and luteolin in aerial parts of* Bulbine capitata* [66], several other antioxidant compounds were identified. Although known antioxidant compounds such as ascorbic acid have been confirmed to promote wound healing, not all the newly identified compounds have been tested for such activity [488–491].

The identified compounds included mainly flavonoids such as flavones and flavonols, flavone and flavonol glycosides, chalcones and dihydrochalcones, and flavonones, although some anthocyanins, proanthocyanidins, and anthrones were also isolated with antioxidant properties. A wide range of plant extracts investigated have been shown to contain flavonoids.* Dorstenia* species are rich in flavonoids some of which are unique to this genus [67, 205], namely, prenylated flavonoids as found in* Dorstenia kameruniana* and twigs of* D. mannii* [206, 207]. Earlier studies have shown that prenylated flavonoids had antioxidant properties, which protected human LDL from oxidation [208]. Those isolated from African medicinal plant extracts were also tested and their antioxidant properties confirmed. The antioxidant activities of three prenylated flavonoids from* D. mannii* (6,8-diprenyleriodictyol, dorsmanin C, 7,8-(2,2-dimethylchromeno)-6-geranyl-3,5,3′,4′-tetrahydroxyflavonol and dorsmanin F, (+)-7,8-[2′′-(1-hydroxy-1-methylethyl)-dihydrofurano]-6-prenyl-5,3′,4′-trihydroxyflavanone) against LDL oxidation and also their free radical scavenging activity have been indicated [187]. Similarly, a diprenylated chalcone, Bartericin A, present in* D. barteri* leaf and twig extracts was shown to have potent antioxidant properties. It was found that this and other prenylated and geranylated chalcones were as active as the prenylated flavones and may account for the anti-inflammatory action of these extracts [276]. Free radical scavenging activity was also confirmed for prenylated anthronoids isolated from the stem-bark of* Harungana madagascariensis *[121] and for proanthocyanidins isolated from the bark of* Burkea africana* [175]. The anti-inflammatory and antioxidant activities of kolaviron, a biflavonoid isolated from a* Garcinia kola* seed extract to scavenge free radicals, which protect against lipid peroxidation and H_2_O_2_-induced DNA strand breaks and oxidized bases, were also reported [114, 116–119, 209]. In addition, the ability of free radical scavenging activity and ability to inhibit lipid peroxidation of Thonningianin A and Thonningianin B, ellagitannins, isolated from* Thonningia sanguinea *have been shown [99, 366]. The anti-inflammatory ability of Griffonianone D ((7E)-(6′′,7′′-dihydroxy-3′′,7′′-dimethyloct-2′′-enyl)oxy-4′-methoxyisoflavone), an isoflavone present in* Millettia griffoniana*, has been established [195]. Prenylated anthronoids, harunmadagascarins A (8,9-dihydroxy-4,4-bis-(3,3-dimethylallyl)-6-methyl-2,3-(2,2-dimethylpyrano)anthrone and B (8,9-dihydroxy-4,4,5-tris-(3,3-dimethylallyl)-6-methyl-2,3-(2,2-dimethylpyrano)anthrone), harunganol B, and harungin anthrone from the stem-bark of* Harungana madagascariensis* have exhibited significant antioxidant activity [121]. Saponins and isofuranonaphthoquinones isolated from different medicinal plant extracts showed antioxidant properties and include the saponin, Balanin 1 (3*β*,12*β*,14*β*,16*β*) cholest-5-ene-3,16-diyl bis (*β*-d-glucopyranoside)-12-sulphate, sterol sulfonated, Balanin 2 (3*β*,2*0S*,2*2R*,2*5R*)-26-hydroxy-22-acetoxyfurost-5-en-3-yl-rhamnopyranosyl-(1→2)-glucopyranoside, and a furostanol saponin isolated from* Balanites aegyptiaca* [104]. Isofuranonaphthoquinones isolated from the roots of* Bulbine capitata*, 5,8-dihydroxy-1-tigloylmethylnaphtho[2,3-c]furan-4,9-dione, 1-acetoxymethyl-8-hydroxynaphtho [2,3-c]furan-4,9-dione, and 1-acetoxymethyl-5,8-dihydroxynaphtho[2,3-c]furan-4,9-dione possess antioxidant activities [68]. Though none of these antioxidant compounds has been directly assessed for wound healing potential, the enhanced wound closure observed with treatment of prenylated flavonoids such as genistein [492] and the demonstrated effect of chalcones on the inflammation process [493] attest to the potential of isolated antioxidants in wound management.

## 4. Crude Extracts of African Medicinal Plants with Confirmed Antioxidant Activities

The antioxidant properties of a larger proportion of African medicinal plants listed in Tables 1 and 2 were tested using either aqueous or organic plant extracts. After confirming antioxidant properties, a correlation was proposed between this property and the general groups of antioxidant compounds that are present in these extracts. No further attempts were made to isolate the specific compounds that may have contributed towards this property. Flavonoids in* Aloe barbadensis* [32], chromone glycosides in* A. claviflora* [35], essential oils in* Artemisia abyssinica*, and* Juniperus procera* [79] as well as* Helichrysum dasyanthum*,* H. felinum*,* H. excisum*, and* H. petiolare* [94], proanthocyanidins in* Burkea africana* bark [175], polyphenols in extracts of* Crataegus monogyna* [321], saponins, and alkaloids in extracts of* Leucosidea sericea *[210, 211] are all considered as major compounds that have contributed to the antioxidant properties of these plants. Reports on a number of* Barleria* species, which includes* B. albostellata, B. greenii*, and* B. prionitis*, have indicated their anti-inflammatory [212] and antioxidant capacities [213]. Unlike the isolated compounds, most of the plants listed for possessing antioxidant activity, including extracts of* Agerantum conyzoides*,* Euphorbia hirta, Kigelia africana, and Nauclea latifolia, *have been shown to possess wound healing ability [494–496].

Furthermore, studies have focused on screening a vast number of plants, used in a specific region, so as to determine their antioxidant properties, Mali [357], South Africa [19, 188, 267, 364], Cameroon [182, 313], Algeria [85], Ghana [98], Burkina Faso [266], Madagascar [23], and Mauritius [293], and anti-inflammatory properties, South Africa [168, 264, 374, 376] and West Africa [400].

## 5. Discussion and Conclusion

The use of traditional herbal remedies as alternative medicine plays a significant role in Africa since it features extensively in primary health care. The search for natural antioxidants, especially from plant sources, as a potential intervention for treatment of free radical mediated diseases is an important research field, especially for those in developing countries. Many polyphenols, including phenolic acids, flavonoids (anthocyanins and anthoxanthins), tannins, and lignans, are known to act as antioxidants and protect against various pathological conditions such as coronary artery disease and wounds, in addition to their anti-inflammatory, antimicrobial, and anticancer activities [214–216].

Flavonoids are a large group of compounds containing several hydroxyl groups on their ring structures and include isoflavonoids and isoflavonoid glycosides, flavones, and flavone glycosides, flavonols and flavonol glycosides, anthocyanins, chalcones and dihydrochalcones, aurones, flavonones and dihydroflavonols, and flavans and biflavonyls. To date, approximately 9000 different flavonoids have been identified from plant sources [217]. Great interest has been dedicated to the antioxidant properties of flavonoids that may function as potent free radical scavengers, reducing agents, and protectors against peroxidation of lipids [208, 218]. Reviews have been published documenting numerous studies on antioxidant efficacy of flavonoids and phenolic compounds as well as on the relationship between their antioxidant activities, as hydrogen donating free radical scavengers, in relation to their chemical structures. The importance of the unsaturation in the C ring of quercetin compared to catechin in the increased antioxidant activity of the former has been presented [216, 219–223]. Also, the importance of the position and number of hydroxyl groups on the phenolic rings in increasing or decreasing the antioxidant properties of these compounds has been emphasized [216, 219–223].

Although many flavonoids have been isolated from different African medicinal plant extracts, the structure-activity relationship of these compounds has not yet been investigated. Recent studies have also shown that some flavonoids are modulators of proinflammatory gene expression, thus leading to the attenuation of the inflammatory response [224]. Examples of these include the lipophilic flavones and flavonols 5,7-dihydroxy-2′,3′,4′,5′-tetramethoxyflavone, 5,4′-dihydroxy-7,2′,3′,5′-tetramethoxyflavone, and 5,7,4′-trihydroxy-2′,3′,5′-trimethoxyflavone isolated from* Psiadia punctulata* [225] and Dinklagin B and C isolated from* Dorstenia dinklagei* [226]. Isolated flavone and flavonol glycosides include kaempferide 3-O-beta-xylosyl (1→2)-beta-glucoside, kaempferol 3-O-alpha-rhamnoside-7,4′-di-O-beta-galactoside, kaempferol 3,7,4′-tri-O-beta-glucoside and quercetin 3-O-[alpha-rhamnosyl (1→6)] [beta-glucosyl (1→2)]-beta-glucoside-7-O-alpha-rhamnoside from* Warburgia ugandensis*, and quercetin-7,4′-disulphate from* Alchornea laxiflora *[159]. Flavanones and dihydroflavonols include dorsmanin I and J and epidorsmanin F and G isolated from* Dorstenia mannii* [227] and Dinklagins A, isolated from the twigs of* Dorstenia dinklagei* [226] and two flavones isolated from the twigs of* Eriosema robustum* [182] and 1*α*,3*β*-dihydroxy-12-oleanen-29-oic (1), 1-hydroxy-12-olean-30-oic acid (2), 3,30-dihydroxyl-12-oleanen-22-one (3), and 1,3,24-trihydroxyl-12-olean-29-oic acid (4), a new pentacyclic triterpenoid (1*α*, 23-dihydroxy-12-oleanen-29-oic acid-3*β*-O-2,4-di-acetyl-l-rhamnopyranoside) (5) from* Combretum imberbe *[138]. Anthocyanins isolated include the cyanidins 3-*O*-(2′′-galloyl-*β*-galactopyranoside) and 3-*O*-(2′′-galloyl-6′′-*O-α*-rhamnopyranosyl-*β*-galactopyranoside) from* Acalypha hispida* [228] and cyanidin 3-*O-β*-D-glucopyranoside and cyanidin 3-*O*-(2-*O-β*-D-xylopyranosyl)-*β*-D-glucopyranoside from* Hibiscus sabdariffa* [266]. When revising the literature, it became apparent that even though most of these medicinal plants and compounds have confirmed antioxidant activity, not many of them have been screened for wound healing potential. As there is an association between antioxidative therapy and wound healing, research in this direction is as imminent as it is important. Furthermore, structure-activity studies on the isolated compounds from African medicinal extracts will be of great interest.

Antioxidants may exert their protective effects via different mechanisms at different stages of the oxidation process. There are those that are able to inhibit the production of free radicals via their ability to chelate transition metal ions and those that are able to quench and stabilise free radicals [229, 230]. Additionally, they are further subdivided into categories according to their functions [230]. Such classification of the newly isolated antioxidant compounds from African medicinal plant extracts is warranted to better understand their antioxidant properties.

It should be noted that the antioxidant activity of the extracts and compounds listed in this review was mostly determined using either single assays or* in vitro* analysis. It is therefore possible that some of these extracts and compounds may not show antioxidant activity when alternative testing methods are used. Furthermore, although* in vivo* studies are encouraged, most studies cited used* in vitro* assays. As antioxidant activity* in vitro* does not necessarily translate to activity* in vivo*, due to pharmacokinetic and pharmacodynamic processes that occurs* in vivo,* it is possible that samples may not be active when tested in animals. Activity of such samples should therefore be confirmed using animal models.

Additionally, attempts should be made to identify the compounds responsible for the proven antioxidant properties where not yet done, and in cases where they have been isolated, their wound healing properties should be investigated. If the activity of the compounds and plants identified in this review is confirmed* in vivo*, they could serve as viable sources for the treatment of wounds in future.

## Figures and Tables

**Table 1:** Medicinal plants with confirmed antioxidant activity, shown to contain compounds that are known to have such activity.

**Family and plant name**	**Vernacular name**	**Plant part**	**Country/area**	**Medicinal use and/or experimental validation**	**Compounds isolated**	**Reference**
**Aloaceae**						

*Aloe barbadensis* Mill.	Burn plant, siber, sbar/essouktouri /mar, sbar	Leaf exudate	Algeria, Morocco, Tunisia	Antioxidant activity. Used as laxative, purgative, diuretic, asthma, baldness, cuts, bounds, skin rash.	Flavonoids, two dihydrocoumarin derivatives and two flavone glycosides	[32–34]

*Aloe claviflora* Burch.	Kraal aloe	Leaf exudate	South Africa	Radical scavenging activity and moderate activity in the lipid peroxidation assay	Chromone glycoside	[35, 36]

*A. saponaria* (Ait.) Haw.	Mpelu Mnemvu Soap aloe, African aloe	Leaf exudate	South Africa	Radical scavenging activity and moderate activity in the lipid peroxidation assay	Chromone glycoside	[35, 37]

*A. thraskii* Baker	Dune aloe, ikhala, umhlaba	Leaf exudate	South Africa	Radical scavenging activity and moderate activity in the lipid peroxidation assay	Chromone glycoside	[35, 36]

**Amaranthaceae**						

*Amaranthus caudatus* L.	Tassel flower	Seed; Young shoots	Ethiopia	Antioxidant properties	Tocopherols, phenolic acids	[38–40]

**Anacardiaceae**						

*Anacardium occidentale* L.	Not signalized	Stem-bark	Nigeria	Anti-inflammatory properties.	Agathisflavone, quercetin 3-*O*-rutinoside, quercetin 3-*O*-rhamnoside	[41, 42]

*Lannea edulis* Engl.	Wild Grape	Root-bark	Zimbabwe	Semipolar extracts high activity both as radical scavengers and lipoxygenase inhibitors. Lipophilic extracts inhibitor of 15-lipoxygenase. Used for painful menstruation, urogenital infection, sexually transmitted diseases.	Two alkylphenols (cardonol 7 and cordonol 13) and three dihydroalkylhexenones	[43–45]

*Lannea velutina* A. Rich	Bemmbeyi Raisinier velu, Lannéa velouté	Leaves, bark, root	Mali	Antioxidant properties	Proanthocyanidins	[46, 47]

*Mangifera indica* L.	Mango Mangoro	Leaves, seeds, stem-bark	Benin Burkina Faso	Anti-inflammatory, analgesic, and hypoglycemic effects. Used to treat urogenital infection, tonic, diarrhoea, tooth ache, gingivitis, liver disease, diabetes.	Polyphenolics, flavonoids	[12, 13, 46, 47]

**Apiaceae**						

*Centella asiatica* (L.) Urb.	Gotu kola	Leaves	South Africa	Antioxidant and anti-inflammatory activities. Used for wound healing. Protection against radiation-induced injury. Cardio protective effect. Oral treatment increased antioxidant enzymes.	Quercetin and tetrandrine	[48–55]

**Apocynaceae**						

*Alstonia boonei* De Wild.	Awun, Egbu	Stem-bark Root-bark	Nigeria Ghana	Anti-inflammatory activity. Used for its analgesic and anti-inflammatory properties.	Rutin, Quercetin robinobioside, Kaempferol-3-O-rutinoside, Kaempferol-3-O-robinobioside	[56–59]

*Catharanthus roseus* (L.) G. Don	Madagascar periwinkle kaka poul, karaktè dezosm blan, zèb sosyé	Whole plant	Madagascar	Antioxidant activity and ability to increase antioxidant enzymes. Used for conjunctivitis.	Phenols	[60]

**Arecaceae**						

*Elaeis guineensis* Jacq.	Ori	Nuts	*Ghana* Nigeria	Anti-inflammatory activity. Used to treat rheumatoid arthritis.	3,4 hydroxybenzaldehyde, p-hydroxybenzoic acid, vanillic acid, syringic acid, ferulic acid, carotenoids, *α*-tocopherol	[12, 61]

**Asclepiadaceae**						

*Secamone afzelii* Rhoem.	Ahaban Kroratima	Stem	Central Africa	Antioxidant and anti-inflammatory properties. Used for wound healing.	Flavonoids, caffeic acid derivatives and *α*-tocopherol.	[62–64]

**Asphodelaceae**						

*Bulbine capitata* Poelln.	Scented grass bulbine	Roots Aerial parts	South Africa	Anti-inflammatory and weak antioxidant and free radical scavenging and lipid peroxidation inhibition activities. Knipholone as a selective inhibitor of leukotriene metabolism. Used as a mild purgative and to cure gonorrhoeal infections.	Anthraquinone Knipholone	[65–73]

*Bulbine frutescens* Willd.	Snake flower, cat's tail, burn jelly plant	Leaf juice Roots	South Africa	Anti-inflammatory and weak antioxidant and free radical scavenging and lipid peroxidation inhibition activities. Knipholone is a selective inhibitor of leukotriene metabolism. Used to treat burns, rashes, blisters, insect bites, cracked lips, acne, cold sores, mouth ulcers and areas of cracked skin.	Phenylanthraquinones, Isofuranonaphthoquinones, Gaboroquinones A and B and 4′-O-demethylknipholone-4′-O-beta-D-glucopyranoside, and Knipholone (anthroquinone)	[65, 67, 70, 74, 75]

*Kniphofia foliosa* Hochst.	Red-not-peker		Kenya	Anti-inflammatory and weak antioxidant and free radical scavenging and lipid peroxidation inhibition activities. Knipholone as a selective inhibitor of leukotriene metabolism. Used for abdominal cramps, wound healing	Anthraquinone: Knipholone	[65, 76–78]

**Asteraceae**						

*Artemisia abyssinica* Sch.Bip.	Chikugn (Amharic) *Arrtta bera (Or)*	Whole plant	Ethiopia	Radical scavenging and antioxidant activities. Used for stomach pain and wound healing.	Essential oils and flavonoids	[79–82]

*A. afra* Jacq. ex Willd.	African wormwood Wild wormwood	Roots, stems and leaves	Ethiopia South Africa	Radical scavenging and antioxidant activities. Used for stomach pain, coughs, colds, fever, loss of appetite, colic, headache, earache, intestinal worms to malaria.	Essential oils and flavonoids	[79, 82–84]

*A. arvensis* L.	Mugwort Wormwood	Whole plant	Algeria	Radical scavenging and antioxidant activities.	Phenolic compounds and flavonoids.	[85]

*A. campestris* L.	Field sagewort Field wormwood	Whole plant	Algeria	Radical scavenging and antioxidant activities. Used to treat insomnia	Phenolic compounds and flavonoids.	[85–87]

*Bidens pilosa* L.	Black jack	Leaves Roots	South Africa	Antioxidant and anti-inflammatory, antibacterial, antihypertensive activities. Used to treat diabetes and backache.	Phenolic compounds: quercetin 3-O-rabinobioside, quercetin 3-O-rutinoside. Two novel methoxylated flavone glycosides: quercetin 3,3′-dimethyl ether 7- O-c¢-L-rhamnopyranosyl-(1 ~ 6)-fl-D-glucopyranoside and the known quercetin 3,3′-dimethyl ether 7-O-fl-D- glucopyranoside	[19, 88–91]

*Cynara scolymus* L.	Globe artichoke	Leaves	Ethiopia	Antioxidative and lipid-lowering properties and eNOS up-regulating ability. Used to treat chronic liver and gall bladder diseases, jaundice, hepatitis and atherosclerosis.	Polyphenolic flavonoid compounds	[14, 15, 92, 93]

*Helichrysum dasyanthum* Sweet	Afrikaans common name of kooigoed (bedding material)	Leaves	South Africa	Antioxidant, radical scavenging and anti-inflammatory activities. Used to treat wounds, infections, respiratory conditions.	Essential oils	[94–96]

*H. petiolare* Hilliard & B.L. Burtt.	Everlasting, Imphepho	Leaves	South Africa	Antioxidant, radical scavenging and anti-inflammatory activities. Used to treat wounds, infections, respiratory conditions, asthma, chest problems and high blood pressure	Essential oils	[94–96]

*Tagetes minuta* L.	Khaki bush stinking roger muster John Henry, wild marigold	Leaves	Madagascar	Antimicrobial and antioxidant activity. Used as anthelmintic, antispasmodic, purgative and for the treatment of gastritis, indigestion and internal worms.	Essential oils.	[23, 97]

**Balanophoraceae**						

*Thonningia sanguinea* Vahl.	Nkomango	Roots	Ghana	Antioxidative and radical scavenging activities and lipid peroxidation inhibitory activity. Used for bronchial asthma, rheumatoid arthritis, atherosclerosis and diabetes.	Ellagitannins: Thonningianin A and B	[98–103]

**Balanitaceae**						

*Balanites aegyptiaca* (L.) Delile	Hausa: aduwa Desert date	Bark and roots	East Africa	Antioxidant properties *in vitro* confirmed. The bark and roots are used as laxatives, and for colic. The bark is used for sore throats, and as a remedy for sterility, mental diseases, epilepsy, yellow fever, syphilis, and tooth aches.	Coumarins, flavonoids, saponins (Balanin 1 (3*β*,12*β*,14*β*,16*β*) cholest-5-ene-3,16-diyl bis (*β*-d -glucopyranoside)- 12-sulphate, a new sterol sulfonated and Balanin 2 (3*β*,2*0S*,2*2R*,2*5R*)-26-hydroxy-22-acetoxyfurost-5-en-3- yl-rhamnopyranosyl-(1→2)-glucopyranoside, a novel furostanol saponin)	[11, 104–106]

**Bignoniaceae**						

*Jacaranda mimosaefolia* D.Don.	Sharpleaf Jacaranda	Leaves Stem-bark	Nigeria	Shown to have antimicrobial activity and used to treat infections	Phenylethanoid glucoside, jacaranone	[107–109]

*Spathodea campanulata* P.Beauv.	African tulip	Stem-bark	Nigeria, Ghana, Cameroon (Yaounde region)	Anti-inflammatory, antioxidant, hypoglycemic, anticomplement and anti-HIV activities. Used to treat itching, arthritis, and diabetes.	Flavonoids and caffeic acid derivatives	[63, 110]

*Tecoma stans* (L.) H.B. & K.	Yellow trumpet bush	Leaves Stem-bark	Nigeria	Anti-diabetic activity is shown.	4-O-E-caffeoyl-alpha-L-rhamnopyranosyl-(1′→ 3)-alpha/beta-D-glucopyranose, E/Z-acetoside, isoacetoside	[107, 111]

**Capparaceae**						

*Cleome arabica* L.	Cleome efeina	Leaves	Egypt	Antioxidant activity, inhibited lipoxygenase activity and calcium ionophore-stimulated LTB4 synthesis in human neutrophils. Used to treat wounds and prevent inflammation	Rutin and quercetin.	[112, 113]

**Clusiaceae**						

*Garcinia kola* Heckel	Bitter cola/aku ilu, agbu ilu. Nigeria Hausa: Góórò *pl.* gwârráá *or* gòòràrràkáí	Seeds	Nigeria	Inhibit lipid peroxidation and protective against H_2_O_2_-induced DNA strand breaks and oxidized bases. Used for laryngitis, coughs, liver disease, bronchitis and throat infections. Inhibits Aflatoxin B1 induced genotoxicity.	Biflavonoid: kolaviron	[114–120]

*Harungana madagascariensis* Poir.	Otori	Stem-bark	Eastern Nigeria	Significant antioxidant activity. Used to treat skin diseases.	Prenylated Anthronoids: harunmadagascarin A [8,9-dihydroxy-4,4-bis-(3,3-dimethylallyl)-6-methyl-2,3-(2,2-dimethylpyrano)anthrone], harunganol B	[121–123]

*Hypericum carinatum* Griseb.	Not signalized	Leaves	Egypt	Antioxidant and radical scavenging activities.	Benzophenones: cariphenone A (6-benzoyl-5,7-dihydroxy-2,2,8-trimethyl-2H-chromene) and cariphenone B (8-benzoyl-5,7-dihydroxy-2,2,6-trimethyl-2H-chromene).	[124, 125]

*H. perforatum* L.	Common St.-Johns' wort	Whole plant	Egypt	Anti-inflammatory and anti-oxidant activities. Free radical scavenging, metal-chelation, and reactive oxygen quenching activities. Protective against scopolamine-induced altered brain oxidative stress status and amnesia in rats. Ability to suppress the activities of 5-lipoxygenase (5-LO) and cyclooxygenase-2 (COX-2), key enzymes in the formation of proinflammatory eicosanoids from arachidonic acid (AA). Analgesic, antiseptic, antispasmodic, digestive, diuretic and sedative.	Flavonoids: Rutin, hyperoside, isoquercitrin, avicularin, quercitrin, and quercetin.	[124, 126–131]

**Cochlospermaceae**						

*Cochlospermum tinctorium* A.Rich.	N'tiribara	Roots	Sudan, Uganda West Africa	Antioxidant activity. Used for malaria, jaundice.	Polyphenols: gallotannins and ferulic acids	[35]

**Combretaceae**						

*Combretum woodii* Drum.	Large-leaved forest bushwillow	Leaf	South Africa	Antioxidant and antibacterial activities. Also tannins showed inhibitory effect on Fe^2+^-induced lipid peroxidation and radical scavenger activity. Used for pneumonia, syphilis, abdominal pain and conjunctivitis.	Polyphenols: Combretastatin B5 (2′,3′4-trihydroxyl,3,5,4′-trimethoxybibenzyl). Tannins.	[132–137]

*Combretum imberbe*	Not signified		South Africa	Combretum species are widely used for treating abdominal disorders (e.g. abdominal pains, diarrhea) backache, bilharziasis, chest coughs, colds, conjunctivitis, dysmenorrhoea, earache, fattening babies, fever, headache	1*α*,3*β*-dihydroxy-12-oleanen-29-oic, 1-hydroxy-12-olean-30-oic acid, 3,30-dihydroxyl-12-oleanen-22-one, and 1,3,24-trihydroxyl-12-olean-29-oic acid, a new pentacyclic triterpenoid (1*α*,23-dihydroxy-12-oleanen-29-oic acid-3*β*-O-2,4-di-acetyl-l-rhamnopyranoside)	[138]

*Guiera senegalensis* J.F.Gmel.	N'kundjè	Leaf	Western Africa	Antioxidant and radical scavenging activities. Used to treat dysentery, diarrhoea, gastro-intestinal pains and disorders, rheumatism, diabetes and fever.	Flavonol aglycones, flavonol glycosides and flavonoids (catechin, myricitrin, rutin and quercetin) as well as tannins (galloylquinic acids (hydrolysable tannins).	[139–143]

*Terminalia sericea* Burch. ex DC.	Silver cluster-leaf	Bark	South Africa	Radical scavenging and antioxidant activities. Used to treat diabetes and pneumonia and to relieve colic	Pentacyclic triterpenoids Anolignan B	[21, 136, 144]

**Commelinaceae**						

*Commelina diffusa* Burm.f.	Wandering Jew Climbing day flower	Leaves	Ghanna	Anti-inflammatory and antioxidant properties. Used to treat fever and is diuretic	Flavonoids	[63, 145]

*Palisota hirsuta* K.Schum.,	Not signified	Aqueous leaf extracts	Nigeria	Anti-inflammatory effects against carrageenan induced hind paw oedema	Not identified	[146, 147]

**Crassulaceae**						

*Bryophyllum* *pinnatum* (Lam.) Oken Synonym: *Kalanchoe pinnata* (Lam.) Pers.	Ufu ivo	Leaves	Nigeria, South Africa	Anti-inflammatory properties. Used for earache.	Flavonoids, polyphenols, triterpenoids	[12, 148, 149]

**Cupressaceae**						

*Juniperus procera* Hochst ex. Endl.	African Juniper	Young twigs and buds	Ethiopia	Antioxidant and free radical scavenging activities. Used to relieve stomach pain.	Essental oils	[79, 150, 151]

**Dioscoreaceae**						

*Dioscorea dumetorum* (Kunth) pax	Yam	Tubers	Nigeria	Antioxidant activity to modify serum lipid and anti-inflammatory activity. Used to treat diabetes.	Dioscorea and Dioscoretine	[152–154]

*Drosera madagascariensis* (DC.) *D. ramentacea* Burchell	Sundew	Roots and flowers	Madagascar	Anti-inflammatory effects. Used to treat coughs and asthma	Flavonoids: hyperoside, quercetin and isoquercitrin	[155, 156]

*Drosera rotundifolia* L.	Round-leaf Sundew	Roots and flowers	Madagascar	Anti-inflammatory effects. Used to treat coughs and asthma	Flavonoids: hyperoside, quercetin and isoquercitrin	[155, 157]

**Euphorbiaceae**						

*Alchornea laxiflora* (Benth) Pax & K. Hoffm.	Wild banana	Leaf and root	Nigeria	Antioxidant and anti-microbial activity. Used to treat jaundice and liver disorders. Also used in food preservation.	Quercetin-7,4′-disulphate, quercetin, quercetin-3′,4′-disulphate, quercetin-3,4′-diacetate, rutin and quercetrin	[158–161]

*Bridelia ferruginea* Benth.	Ora	Leaves, stem and bark	West Africa Democratic republic of Congo, Nigeria	Anti-inflammatory. Used to treat diarrhea, dysentery, gastro-intestinal disorders, gynecological disorders (including sterility), and rheumatic pains.	A bioflavonoid: Gallocatechin-(4′→O →7)-Epigallocatechin.	[12, 57, 162–166]

*Mallotus oppositifolius* (Geiseler) Muell. Arg.	Jororo Káfàr mútúwàà Senampendi Mvundza jembe	Leaves, roots	West Africa Nigeria	Antioxidant, anti-inflammatory and antimicrobial activities. Used for abortion.	Flavonoids: quercetin and quercitrin.	[167–172]

**Fabaceae**						

*Aspalathus linearis* (Brum. F.) R. Dahlgr.	Rooibos	Leaves	South Africa	Radical Scavenging Capacity Used to treat stomach cramps, insomnia, and to reduce stress.	Phenolic Fractions, Tannins and monomeric flavonoids aspalathin, nothofagin, quercetin, rutin, isoquercitrin, orientin, isoorientin, luteolin, vitexin, isovitexin, and chrysoeriol.	[16–21, 173, 174]

*Burkea africana* Hook	Wild Syringa	Bark	Mali and Sub-Saharan Africa	Antioxidant and radical scavenging activity. Used to treat coughs, colds, stomach obstruction, infusions against gonorrhoea and syphilis.	Proanthocyanidins; fisetinidol-(4alpha- >8)-catechin 3-gallate and bis-fisetinidol-(4alpha- >6, 4alpha- >8)-catechin 3-gallate, with smaller amounts of flavan-3-ols (catechin, epicatechin and fisetinidol)	[175, 176]

*Crotalaria podocarpa* DC.	Crotalaria	Roots	South Africa	Anti-inflammatory activity. Used for the treatment of sore-eyes and boils. Expectorant.	Flavonoids	[67, 177]

*Cyclopia intermedia* E. Mey. and *C. subternata* Vog.	Honeybush	Leaves and stem	South Africa	Antioxidant activity. Used as tonic for colds, catarrh and tuberculosis.	Pinitol, shikimic acid, p-coumaric acid, 4-glucosyltyrosol, epigallocatechin gallate, the isoflavone orobol, the flavanones hesperedin, narirutin and eriocitrin, a glycosylated flavan, the flavones luteolin, 5-deoxyluteolin and scolymoside, the xanthone mangiferin and the flavonol C-6-glucosylkaempferol. Phenolic content: tyrosol and a methoxy analogue, 2-[4-[O-alpha-apiofuranosyl-(1′′→6′)-beta-d-glucopyranosyloxy] phenyl]ethanol, 4-[O-alpha-apiofuranosyl-(1′′→2′)-beta-d-glucopyranosyloxy]benzaldehyde, five glycosylated flavonols, two isoflavones, four flavanones, two isoflavones, and two flavones	[19, 21, 178–181]

*Eriosema robustum*		Twigs	Burundi, Ethiopia, Kenya, Rwanda, Tanzania, Uganda, Democratic Republic of Congo and Cameroon	Used traditionally for the treatment of coughs in East Africa and skin diseases in Central Africa	2′,3′,5′,5,7-pentahydroxy-3,40-dimethoxyflavone, 2′,3,5′,5,7-pentahydroxy-4′-methoxyflavone	[182, 183]

*Erythrina latissima* E. Mey.	Broad-leaved coral tree	Stem Wood Root wood Seeds	South Africa Botswana	Antimicrobial activity and weak radical scavenging properties. Purgative.	Flavonoids and isoflavonoids. Isoflavones: erylatissin A and B. Flavanone: erylatissin C and flavonoids and Isoflavone glycosides: 4′-hydroxyisoflavone-7-O-beta-D-glucopyranoside (compound 1); 4′-hydroxyisoflavone-7-O-alpha-L-rhamnosyl (1→6)-beta-D-glucopyranoside (compound 2); and a new compound 4′, 8-dimethoxy isoflavone-7-O-alpha-L-rhamnosyl (1→6) glucopyranoside (8-O-methylretusin-7-O-alpha-L-rhamnosyl (1-6)-beta-D-glucopyranoside) (compound 3) Isoflavonoids: 5,7-dihydroxy-2′,4′,5′-trimethoxyisoflavanone.	[67, 184–186]

*E. lysistemon* Hutch.	Common coral tree; lucky bean tree	Bark	South Africa	Mild antioxidant activity. Used to treat sores, wounds, abscesses and arthritis.	Three prenylated flavonoid derivatives; 5,7,4′-trihydroxy-8-(3′′′-methylbut-2′′′-enyl)-6-(2′′-hydroxy-3′′-methylbut-3′′ enyl) isoflavone (isoerysenegalensein E), 5,7,2′-trihydroxy-4′-methoxy-5′-(3′′-methylbut-2′′-enyl) isoflavanone (lysisteisoflavanone), 5, 4′-dihydroxy-6-(3′′′-methylbut-2′′′-enyl)-2′′-hydroxyisopropyl dihydrofurano [4′′,5′′:8,7] isoflavone (isosenegalensin), together with the four known flavonoids abyssinone V-4′-methylether, alpinumisoflavone, wighteone and burttinone	[187–190]

*Melilotus elegans* Salzm. ex Ser. (syn. *M.* *abyssinica* Baker)	Egug, Gugi, Yemen berri Elegant sweet clover	Leaves	Ethiopia	Anti-inflammatory properties. Used for asthma, haemorrhoid, wounds, excavated sore, piles, ulcers mouth infection, lacerated wounds, haemorrhoids, bronchial asthma (personal communication)	Flavonoids: kaempferol	[191–194]

*Millettia griffoniana* *Baill.*	Not signalized	Root-bark and seeds	Cameroon	Anti-inflammatory activity. Used as an antimalarial.	Coumarin: 4-hydroxy-3-(3′,4′-methylenedioxyphenyl)-5,6,7-trimethoxycoumarin, durmillone, odorantin, 7-methoxyebenosin, calopogonium isoflavone B and 7,2′-dimethoxy-4′,5′-methylenedioxy isoflavone maximaisoflavone G (5) and 7-hydroxy-6-methoxy-3′,4′-methylenedioxyisoflavone and new prenylated isoflavonoids griffonianones A, B, C, D and E.Griffonianone D ((7E)-(6′′,7′′-dihydroxy-3′′,7′′-dimethyloct-2′′-enyl)oxy-4′-methoxyisoflavone), an isoflavone.	[195–202]

*Parkia biglobosa* (Jacq.) Benth	African Locust Bean Nèrè Ojinyi	Bark Seeds	Mali Sudan Ivory Coast	Anti-inflammatory activity. Used as antiseptic and to treat coughs, chest pain, and wound healing	Tocopherol, ascorbic acid (Seeds)	[12, 33, 34, 36–39, 43–53, 55, 64, 66–72, 118, 119, 121, 138, 159, 182, 195, 203–235]

*Peltophorum africanum* Sond.	Weeping wttle	Root and bark	South Africa	Antioxidant and antibacterial activities Used to treat diarrhoea, dysentery, sore throat, wounds, back and joint pains, HIV-AIDS, venereal diseases and infertility.	Flavonol glycosides and flavonol glucoside gallates	[236–238]

*Piliostigma thonningii* (Schum.) Milne- Redh	Camel's foot tree, Monkey Bread Niama (Mali). Abefe Kalgo Okpoatu Omepa	Root, bark, pods, leaves	Nigeria, Ethiopia Botswana, Kenya, Namibia, Senegal, South Africa, Sudan, Tanzania, Uganda, Zambia	Anti-oxidant and anti-inflammatory properties. Used to treat wounds, chronic ulcers, cough, respiratory disorders and toothache, gum inflammation, arthritis, headache, backache, and antioxidant supplement.	Proanthocyanidins epicatechin, catechin trimers and oligomers, flavonoids, polyphenolics, C-methylflavonols (in the leaf extract)	[12, 58, 239–245]

*Sutherlandia frutescens* R.Br.	Cancerbush Phetola	Leaves	South Africa	Superoxide and hydrogen peroxide scavenging activities. Used as tonic to boost the immune system.	Canavanine, pinitol	[246–248]

*Trigonella foenumgraecum* L.	Fenugreek	Seeds	Ethiopia, Morocco	Protective effect against Oxidative stress during ischemia-reperfusion. It is hypolipidemic, and is also used to treat boils and to improve appetite.	Free phenolics and Vit C.	[26–28, 249, 250]

**Humiriaceae**						

*Sacoglottis gabonensis* Urb.	Cherry tree, ozouga	Stem-bark	West Africa	Antioxidant activity.	Bergenin	[251–254]

**Hypoxidaceae**						

*Hypoxis hemerocallidea* Fisch. & C.A. Mey.	African potato	Corms	South Africa	Antioxidant activity. Used to treat tuberculosis, cancer, bladder disorders, benign prostatic hyperplasia.	Rooperol	[188, 255–257]

**Lamiaceae**						

*Ocimum basilicum* L.	Mükandu Basil	Leaves	Burkina Faso Ethiopia	Intermediate antioxidant activity and high antibacterial activity. Used in Ethiopia to treat Conjunctivitis and in Kenya to treat colds and stomacheache.	Linalool basil oil Methyl chavicol, eugenol, (E)-methyl cinnamate, thymol, linalool	[23, 258]

*Ocimum gratissimum* L.	Tea bush, Scent leaf/Nchuanwu. Ujuju okpevu Basil	Leaves	Popular republic of Congo (ex Brazaville Congo) Eastern Nigeria	Antioxidant activity Popular republic of Congo it is used as a laxative, purgative, and to treat snakebite, diabetes, tooth ache, gingivitis.	Xanthomicrol, cirsimaritin, rutin, kaempferol 3-*O*-rutinoside and vicenin-2 were identified as the major flavonoids, whereas luteolin 5-*O*-glucoside, luteolin 7-*O*-glucoside, apigenin 7-*O*-glucoside, vitexin, isovitexin, quercetin 3-*O*-glucoside and isothymusin were detected as minor constituents.	[12, 58, 258–262]

**Lauraceae**						

*Cinnamomum zeylanicum* Breyne	Cinnamon leaf	Leaves	Madagascar Ethiopia	Very high antioxidant and high antimicrobial activities. Used to treat diarrhoea, rheumatism, colds and hypertension	Cinnamaldehyde, eugenol and eugenyl acetate to be the main constituents of cinnamon oil.	[22–24, 263]

*Ocotea bullata* (Burch.) Baill.	Black stinkwood Unukane (Zulu)	Bark	South Africa	Anti-inflammatory, cyclooxygenase inhibitory activity. Urinary disorders, headaches.	Monoterpenoids	[188, 264]

*Ravensara aromatica* Sonn.	Nutmeg havozo	Bark Leaf	Madagascar	Low antioxidant and antimicrobial activity. Useful for chronic respiratory conditions, and sometimes helpful in cases of asthma.	Essential oils, principally composed of the monoterpene hydrocarbons a-pinene, sabinene, myrcene, limonene, & the azulene: iso-ledene. In barks, estragole (methyl chavicol) but leaves contain b-myrcene, 1,8-cineole, linalool, and carotol.	[23, 25, 265]

**Malvaceae**						

*Hibiscus sabdariffa* L.	Red tea, sorelle Rosella	Flowers	Nigeria South Africa	Antimutagenic activity and free radical scavenging effects on active oxygen species Used against insomnia, colic.	Flavonol glucoside hibiscitrin Anthocyanins. Such as cyanidin 3-*O-β*-D-glucopyranoside, cyanidin 3-*O*-(2-*O-β*-D-xylopyranosyl)-*β*-D-glucopyranoside, delphinidin 3-*O-β*-D-glucopyranoside and delphinidin 3-*O*-(2-*O-β*-D-xylopyranosyl)-*β*-D-glucopyranoside.	[19, 21, 266–269]

**Meliaceae**						

*Trichilia roka* Chiov.	Soulafinzan	Root	Tropical Africa Mali	Significantly protective against CCl_4_-induced liver damage and prevented perisinusoidal fibrosis. Used to treat malaria, abdominal pain and dermatitis.	Polyphenols	[270, 271]

**Menispermaceae**						

*Sphenocentrum jollyanum* Pierre	Akerejupon ajo	Fruit Root	West Africa	Anti-inflammatory activity. Used to treat inflammatory-based diseases	Furanoditerpenes: columbin, isocolumbin. Flavonoids-rich fraction.	[272–274]

*Tinospora bakis*		Whole plant	Sudan	Anti-inflammatory activity. To treat headache and rheumatism	A diterpenoid furanolactone, columbin	[275]

**Moraceae**						

*Dorstenia barteri* var. *subtriangularis* (Engler) M.E.E.Hijman & C.C.Berg	Contrayerva	Twigs/leaves	Cameroon	Antioxidant properties account for the anti-inflammatory action of these extracts Used to treat arthritis, rheumatism, gout, headache and other forms of body pains.	Prenylated flavonoids: Three diprenylated chalcones: bartericins A (-)-3-(3,3-dimethylallyl)-5′-(2-hydroxy-3-methylbut-3-enyl)-4,2′,4′-trihydroxychalcone, bartericins B (+)-3-(3,3-dimethylallyl)-4′,5′-[2′′′-(1-hydroxy-1-methylethyl)-dihydrofurano]-4,2′-dihydroxychalcone and bartericins C 3,4-(6′′,6′′-dimethyldihydropyrano)-4′,5′-[2′′′,-(1-hydroxy-1-methylethyl)-dihydrofurano]-2′-hydroxychalcone and also two novel diprenylated chalcones: 3,5′-di-(2-hydroxy-3-methylbut-3-enyl)-4,2′,4′-trihydroxychalcone, 3, 4-(2,2-dimethylpyrano)-3′-(2-hydroxy-3-methylbut-3-enyl)-2′,4′-dihydroxychalcone, 4,2′, 4′-trihydoxy-3′-prenylchalcone and 4,2′,4′-trihydoxy-3, 3′-diprenylchalcone; and 5,7,4′-trihydoxy-8-prenylflavone. Other known compounds such as stipulin, 4-hydroxylonchocarpin, kanzonol B, 3′-(2-Hydroxy-3-methylbut-3-enyl)-5′-(3,3-dimethylallyl)-4,2′,4′-trihydroxychalcone, and dorstenone.	[67, 276–281]

*D. ciliata* Engl.	Contrayerva	Aerial parts	Cameroon Central Africa	Antiradical and antioxidant activities. Used as food additive.	phenolic compound (6-prenylapigenin) Flavones: (ciliatin A) 5,4′-Dihydroxy-5′′-isopropenyldihydrfuranol[2′′,3′′:7,6]flavone (ciliatin B) 7,4′-Dihydroxy-3′-methoxy-6′′,6′′-dimethyldihydropyranol[2′′,3′′:5,6].	[282–284]

*D. convexa* De Wild.	Contrayerva	Twigs and leaves	Democratic Republic of the Congo	Antioxidant properties account for the anti-inflammatory action of these extracts. Used to treat arthritis, rheumatism, gout, headache and other forms of body pains.	Prenylated flavonoids	[67, 276, 280]

*D. mannii* Hook.f.	Contrayerva	Twigs/leaves Aerial parts	Central Africa	Antioxidant action against copper-induced LDL oxidation, this activity is like the non-prenylated flavonoid quercetin. Also, inhibition of platelet aggregation and influence of cyclooxygenase and lipoxygenase activity. Used to treat rheumatism, stomach disorders. Anti-trichomonal activity.	Grenylated and prenylated flavonoids and flavonones: Flavonones: 6,8-diprenyl-5,7,3′4′-tetrahydroxyflavanone, 4-hydroxylonchocarpin, 4-methoxylonchocarpin, 6-prenylchrysoeriol, 6,8-diprenyleriodictyol, gancaonin P and Prenylated flavonoids: 6,8-diprenyleriodictyol, dorsmanin C 7,8-(2,2-Dimethylchromeno)-6-geranyl-3,5,3′,4′-tetrahydroxyflavone and dorsmanin D 6,8-Diprenyl-3,5,7,4′-tetrahydroxy-3′-methoxyflavone, dorsmanins 1, J and 2′′-epimers of dorsmanins F (6,7-(2,2-dimethylpyrano)-8-prenyl-5,3′,4′-trihydroxyflavanone, G (6,7-(2,2-dimethyldihydro-pyrano)-8-prenyl-5,3′,4′-trihydroxflavanone). Also, dorsmanins F and G. Four new prenylated flavanones, named dorsamine F (7,8-[2′′-(1-hydroxy-1-methylethyl)-dihydrofurano]-6-prenyl-5,3′,4′-trihydroxyflavanone), dorsmaine G (6,7-[2′′-(1-hydroxy-1-methylethyl)dihydrofurano]-8-prenyl-5,3′,4′-trihydroxyflavanone) and dorsamine H (6-prenyl-8-(2-hydroxy-3-methylbut-3-enyl)-5,7,3′,4′-tetrahydroxyflavanone).	[67, 187, 207, 285–287]

*D. poinsettifolia* var. *angusta* Engl.	Dingetenga	Whole plant	Cameroon	Antiradical and antioxidant activities. Used to treat infected wounds.	Grenylated and prenylated flavonoids. The unusual 4-phenyl-substituted dihydrocoumarin and the rare geranyl-and prenyl-substituted Chalcone.	[207, 288, 289]

*D. psilurus* Welw.	Dingetenga	Roots	Cameroon Central Africa	Antiradical and antioxidant activities. Used against snakebite and to treat rheumatism, headache and stomach disorders.	Grenylated and prenylated flavonoids. Three phenolic compounds: 6,8-diprenyl-3′ [O],4′-(2,2-dimethylpyrano)-3,5,7-trihydroxyflavone, 3,6-diprenyl-8-(2-hydroxy-3-methylbut-3-enyl)-5,7,2′,4′-tetrahydroxyflavone and an unusualB/C ring modified flavonoid derivative for which the names dorsilurins C, D and E, respectively, are proposed. Two new flavones, dorsilurins A and B, and a new benzofuran derivative have been isolated from Dorstenia psilurus, together with three known phenylpropanoid derivatives, stearyl-p-coumarate [octadecanyl 3-(4-hydroxyphenyl)prop-2-enoate], stearyl ferulate [octadecanyl 3-(4-hydroxy-3-methoxyphenyl)prop-2-enoate] and psoralen.	[206, 282, 290–292]

**Myrtaceae**						

*Eugenia elliptica* Sm. *Syzygium smithii* (Poir.) Nied.	Lilly Pilly	Leaves	Mauritius	Modulate the expression of the antioxidant enzyme genes.	Quercetin-3-O-galactoside (hyperoside), kaempferol-3-glucoside (astragalin), quercetin-3-O-glucoside (isoquercitrin), (+)-catech	[293, 294]

*E. fasciculata* Wall.	Not signalized	Leaves	Mauritius	Modulate the expression of the antioxidant enzyme genes.	Quercetin-3-O-galactoside (hyperoside), kaempferol-3-glucoside (astragalin), quercetin-3-O-glucoside (isoquercitrin), (+)-catech. procyanidin B2 dimer and (-)-epicatechin	[293]

*E. orbiculata* Lam.	Not signalized	Leaves	Mauritius	Modulate the expression of the antioxidant enzyme genes.	Quercetin-3-O-galactoside (hyperoside), kaempferol-3-glucoside (astragalin), quercetin-3-O-glucoside (isoquercitrin), (+)-catech. quercetin-3-O-rutinoside (rutin),	[293, 295]

*E. pollicina* J.Gueho & A.J.Scott	Not signalized	Leaves	Mauritius	Modulate the expression of the antioxidant enzyme genes.	Quercetin-3-O-galactoside (hyperoside), kaempferol-3-glucoside (astragalin), quercetin-3-O-glucoside (isoquercitrin), (+)-catech. (-)-epicatechin gallate	[293, 296]

*Monimiastrum acutisepalum* J. Gueho & A.J. Scott.	Not signalized	Leaves	Mauritius	Modulate the expression of the antioxidant enzyme genes.	Quercetin-3-O-galactoside (hyperoside), kaempferol-3-glucoside (astragalin), and quercetin-3-O-glucoside (isoquercitrin). (+)-catechin	[293–295]

*M. globosum* J.Gueho & A.J.Scott	Not signalized	Leaves	Mauritius	Modulate the expression of the antioxidant enzyme genes.	Quercetin-3-O-galactoside (hyperoside), kaempferol-3-glucoside (astragalin), and quercetin-3-O-glucoside (isoquercitrin). (-)-epicatechin gallate	[293]

*Syzygium aromaticum* (L.) Merr. & L.M.Perry	Clove bud	Dried flowers Buds	Madagascar Sudan	Antioxidant and antimicrobial activities. Used to treat tooth ache and throat inflammation.	Eugenol Methyleugenol	[23, 297, 298]

*S. coriaceum* J.Bosser & J.Guého	Bois de pomme		Mauritius	Abilities to modulate the expression of the antioxidant enzyme genes.	Phenols and flavonoids: Quercetin-3-O-rutinoside, kaempferol-3-glucoside (astragalin) and quercetin-3-O-glucoside (isoquercitrin), (+)-catechin, procyanidin B1 dimer, (-)-epicatechin gallate	[293]

*S. glomeratum* DC.	Bois de pomme	Leaves	Mauritius	Abilities to modulate the expression of the antioxidant enzyme genes. Used to treat boils, abscesses, fever and wounds and as expectorant.	Phenols and flavonoids: kaempferol-3-glucoside (astragalin) and quercetin-3-O-glucoside (isoquercitrin), procyanidin B1 dimer, (-)-epicatechin gallate, chlorogenic acid, (-)-epicatechin	[293]

*S. guehoii*	Not signalized		Mauritius	Abilities to modulate the expression of the antioxidant enzyme genes.	Phenols and flavonoids: quercetin-3-O-rutinoside (rutin), kaempferol-3-glucoside (astragalin) and quercetin-3-O-glucoside (isoquercitrin), (+)-catechin, chlorogenic acid, procyanidin B2 dimer	[293]

*S. mauritianum* J.Gueho & A.J.Scott	Not signalized	Leaves	Mauritius	Abilities to modulate the expression of the antioxidant enzyme genes.	Phenols and flavonoids: quercetin-3-O-rutinoside (rutin), kaempferol-3-glucoside (astragalin) and quercetin-3-O-glucoside (isoquercitrin), (+)-catechin, chlorogenic acid	[293]

*S. petrinense* J.Bosser & J.Guého	Not signalized		Mauritius	Abilities to modulate the expression of the antioxidant enzyme genes.	Phenols and flavonoids: quercetin-3-O-rutinoside (rutin), kaempferol-3-glucoside (astragalin) and quercetin-3-O-glucoside (isoquercitrin), procyanidin B1 dimer, chlorogenic acid	[293]

*S. venosum* (Lam.) J.Gueho & A.J.Scott	Not signalized		Mauritius	Abilities to modulate the expression of the antioxidant enzyme genes.	Phenols and flavonoids: quercetin-3-O-rutinoside (rutin), kaempferol-3-glucoside (astragalin) and quercetin-3-O-glucoside (isoquercitrin), (+)-catechin, procyanidin B2 dimer	[293, 295, 299]

**Oleaceae**						

*Olea europaea* subsp *africana* (Mill.)P.S. Green	African wild olive	Leaves	South Africa	Potent antioxidant activity. Used as eye lotions and tonics, lower blood pressure, improve kidney function and deal with sore throats. The early Cape settlers used the fruits to treat diarrhoea	Oleuafricein (mixture of oleanolic acid and ursolic acids), Triterpenoids and oleoropein.	[84, 300, 301]

**Pedaliaceae**						

*Harpagophytum procumbens* DC. ex Meissner	Devil's claw	Root	South Africa Native to the Kalahari Desert of southern Africa, Namibia and Botswana.	Anti-inflammatory and ability to inhibit the expression of cyclooxygenase-2 and inducible nitric oxide by suppression of NF-kappaB activation. Used for pain, muscular tension, osteoarthritis, degenerative rheumatism or painful arthrosis and tendonitis as well as tonic for loss of appetite and dyspeptic complaints.	Roots contain iridoid glycosides mainly harpagoside. Other constituents are flavones and flavonols kaempferol, and luteolin.	[302–312]

**Piperaceae**						

*Piper guineense* Schum. & Thonn.	West African black pepper Bush pepper Ikom, Amana kakwale iyeyeh ashoesie taquale Meshoro	Fruit, seed and leaf	Ghana, West Africa Nigeria Cameroon	Antioxidant activity.	Volatile oil components-monoterpenes, sesquiterpenes, terpenoids, lignans and sterols.	[313–316]

**Podocarpaceae**						

Podocarpus species *Podocarpus* *elongates Podocarpus* *falcatus,* *Podocarpus henkelii* *and Podocarpus latifolius*		Leaves and young stems	Eastern and Southern Africa	These species are used to treat fevers, asthma, coughs, cholera, chest complaints, arthritis, rheumatism, painful joints and venereal diseases	Diterpenoids, bioflavonoids and Totarol	[317]

**Ranunculaceae**						

*Nigella sativa* L.	Black cumin	Seed	African countries in the Mediterranean region	Antioxidant potentials through scavenging ability of different free radicals including the superoxide anion radical, inhibition of lipid peroxidation, and protection of liver against carbon tetrachloride (CCl4)-induced liver fibrosis in rabbits Used to treat diarrhoea, asthma, and as gastroprotective agent.	Oil: Thymoquinone	[29–31, 318, 319]

**Rosaceae**						

*Crataegus monogyna* Jacq.	Hawthorn, May Blossom, May Day Flower, White Thorn.	Fresh vegetative and reproductive organs	Mauritius, Northern Africa	Antioxidant activities. Used for its neuro- and cardiosedative actions.	Polyphenols: (proanthocyanidin, flavonoid, anthocyanin, (-)-epicatechin, procyanidin B2, chlorogenic acid). Flavonoids:quercetin and quercetrin, glycosides, proanthocyanidins, anthocynaidins, saponins, tannins, and cratetegin Also, Vitamin C.	[320–323]

*Leucosidea sericea*		Leaf, bark and roots	Southern Africa	Antimicrobial and anti-inflammatory properties	Phenolics, alkaloids and saponins	[210]

*Pygeum africanum* Hook. f.	African plum tree Red Stinkwood	Bark	South Africa	Anti-inflammatory. Used to treat against benign prostatic hyperplasia, prostatitis	14% triterpenes (urolic acids, oleanolic acid, crataegolic acid), 0.5% n-docosanol Phytosterol (*β*-sitosterol, *β*-sitosterone, Campesterol	[188, 324–327]

**Rubiaceae**						

*Crossopteryx febrifuga* Benth.	Roger Blench “rima jogoo-hi/je”	Seeds Leaf and roots	Mali Nigeria	Radical scavenging and lipoxygenase inhibition activities. Used to treat fever and various respiratory diseases	Flavonoids	[328–330]

**Rutaceae**						

*Agathosma betulina* (Berg.) Pillans.	Round-leaf buchu	Leaves, stems	South Africa	Hydroxyl radical ion scavenging ability. Used for stomach problems, kidney and urinary track diseases.	Essential oils and flavonoids	[188, 331, 332]

*A. crenulata *(L.) Pillans	Oval-leaf buchu	Leaves, stems	South Africa	Anti-inflammatory activity. Used to treat benign prostatic hyperplasia, prostatitis, diabetes, inflammation of the colon, gums, and mucous membranes. Leaves chewed to relieve stomach complaints.	Essential oils and flavonoids	[84, 188, 331, 332]

*Fagara zanthoxyloides* Lam.	xeti, xe Wô	Roots, root-bark	Cameroon, Uganda	Antioxidant activity. Used to treat gingivitis, toothache, urinary and venereal diseases, rheumatism and lumbago, malaria and other infections.	Phenylethanoid derivative, lignans and fagaronine	[333–336]

**Sapindaceae**						

*Dodonaea viscosa* Jacq. Synonyms: *Dodonaea angustifolia* L. f.; *Ptelea viscosa* L.	Umusasa	Leaves	Rwanda	Anti-inflammatory activity by inhibiting the synthesis of prostaglandin (PG) E(2). Used to treat rheumatism, skin infections, diarrhea, stomachaches, pains of hepatic and splenic origin, uterine colic. It is also used as an antipruritic in skin rashes and for the treatment of some throat, dermatitis and hemorrhoids.	Quercetin, isorhamnetin	[337–341]

**Xanthorrhoeaceae**						

***Aloe ferox Mill.***	Bitter aloe or Cape aloe	Leaves	South Africa, Lesotho	*A. ferox* gel contains at least 130 medicinal agents with anti-inflammatory, analgesic, calming, antiseptic, antiviral, antiparasitic and anticancer effects	Chromones, anthraquinones, anthrone, anthrone-C-glycosides, and other phenolic compounds Barbaloin	[9]

**Zingiberaceae**						

*Siphonochilus aethiopicus* (Schweinf.) B.L. Burtt.	Wild ginger Natal ginger African Ginger	Rhizome	South Africa	Anti-inflammatory activity through cyclooxygenase inhibitory (prostaglandin-synthetase inhibition), activity. Used to treat Coughs, colds, asthma.	Sesquiterpenoid	[188, 264, 342]

**Table 2:** Medicinal plants with confirmed antioxidant activity or medicinal plants that contain compounds that are not known to have antioxidant activity.

**Family and plant name**	**Vernacular name**	**Plant part**	**Country/area**	**Medicinal use and/or experimental validation**	**Compounds isolated**	**Reference**
**Acanthaceae**						

*Barleria species* * B. albostellata,* * B. greenii,* *B. prionitis*		Leaves, twigs and roots	South Africa	Anti-inflammatory and antioxidant activities	Not identified	[212, 213]

*Hypoestes rosea* Decne.	Not signalized	Leaf extract	Nigeria	Anti-inflammatory activity due in part to its ability to inhibit NF-kappaB activation through direct inhibition of IkappaB kinase (IKK).	Diterpene: Hypoestoxide (a bicyclo [9,3,1] pentadecane)	[380, 381]

**Aizoaceae**						

*Glinus lotoides *L.	“Mettere” Hairy carpet -weed	Seeds	Cameroon Ethiopia, Sudan, Uganda, Egypt.	Used to treat cardiovascular and gastrointestinal system.	Three flavonoids: apigenin-7-O-glucoside, isovitexin, and luteolin-7-O-glucoside Three isoflavonoids: 5,7,2′,4′-tetrahydroxy-6-(3,3-dimethylallyl)isoflavone, 5,7,4′-trihydroxy -6,3′-di-(3,3-dimethylallyl)isoflavone, and 5,7,2′,4′-tetrahydroxy-6,3′-di-(3,3-dimethylallyl) isoflavone.	[290, 382–386]

*G. oppositifolius *(L.) Aug. DC.	Balasa	Whole plant	Mali	Antioxidant and radical scavenging abilities.	kaempferol 3-O-galactopyranoside	[387, 388]

**Aloaceae**						

*Aloe claviflora* Burch.	Kraal aloe		South Africa	Free radical scavenging and moderate inhibition in lipid peroxidation. Used as a purgative.	Not identified	[35]

*A. maculata* Forssk. (=*A. saponaria*)	“Yellow Form” Tiger Aloe, Soap Aloe		South Africa	Free radical scavenging and moderate inhibition in lipid peroxidation. Used as a purgative.	Not identified	[35]

*A. thraskii *Baker	Dune aloe		South Africa	Free radical scavenging and moderate inhibition in lipid peroxidation. Used as a purgative.	Not identified	[35]

**Anacardiaceae**						

*Sclerocarya birrea *(A.Rich.) Hochst	Marula	Stem-bark		Anti-inflammatory activity. Used to treat diabetes, tonsillitis, snake bite and also diarrhoea.	Not identified	[389]

**Annonaceae**						

*Enantia chlorantha* Oliver	Erenbavbogo, Mföl Muamba	Root, stem-bark	Nigeria	Anti-inflammatory activity. Used to treat ulcers and leprous spots wounds. Bark sap is taken as decoction against diarrhoea.	Not identified	[390–393]

*Uvaria afzelii* Sc. Elliot	Pareho-houon, Bahie oulin	Leaves, roots and stem-bark	Ivory Coast	Used as for its antiparasitic activity	Anthocyanins and other flavonoids	[394–396]

*U. chamae* P.Beauv.	Okandii Anweda tsoGa	Stem, bark Leaves, root	Ivory Coast Nigeria	Used for its antiplasmodial activity.	Polyphenols	[12, 397, 398]

**Apocynaceae**						

*Picralima nitida* Th. & H. Dur.	Ghana: Kpetepetetso, Kanwini, Kanwinu Cameroon: *motoko-toko*	Seeds Stem-bark	Ghana	Anti-inflammatory activity. Used for its analgesic and anti-inflammatory properties.	Not identified	[168, 399–402]

*Rauvolfia vomitoria* Afzel.	Asofeyeje, adapopo Mwanje	Root-bark	Ghana	Anti-inflammatory activity. Used for its analgesic, antipyretic and anti-inflammatory activities. Also to treat scabies, high blood pressure, fever and snakebites.	Not identified	[56]

**Araliaceae**						

*Cussonia barteri* Seem.	Cabbage tree	Leaves Roots	Nigeria, Mali	Antioxidant and radical scavenging abilities. Inhibitory activity on 5-lipoxygenase and cyclooxygenase-1.	Not identified	[357, 403]

**Arecaceae**						

*Hyphaene thebaica* Mart.	Not signalized	Shell	Niger	Antioxidant activity	Not identified	[11]

**Asclepiadaceae**						

*Calotropis procera *(Aiton) W.T.Aiton	African milk weed Sodom apple/Giant milkweed/	Latex	Ethiopia	Anti-inflammatory and antioxidant activities.	Not identified	[404]
	Swallow-wort/Auricula tree.		Sudan	Used to control dermal fungal infections and for pain relief. Latex used against scorpion stings and roots for jaundice.		

*Gongronema latifolium* Benth.	Not signalized	Leaves	Nigeria	Antioxidant activity	Not identified	[405–407]

*Leptadenia hastata* Decne.	Not signalized	Leaves	Niger	Antioxidant activity	Not identified	[11]

*Pachycarpus rigidus* E. Mey.	Not signalized	Bark	South Africa	Antioxidant activity. Used to treat pain in the joints	Not identified	[188]

**Asparagaceae**						

*Asparagus virgatus* Baker Refug. Bot. (Saunders)	Broom asparagus	Bark	South Africa	Antioxidant activity. Used to treat syphilis, anthelmintic	Not identified	[35]

**Asteraceae**						

*Ageratum conyzoides* L.	Inkuruba Herbe à bouc	Whole plant	Central Africa, Rwanda Ethiopia	Antioxidant and anti-inflammatory properties. Used to treat mastitis and urogenital infections and to dress wounds. Also as a gastroprotective.	Not identified	[12, 408, 409]

*Artemisia herba-alba*	Desert wormwood, shih	Aerial parts	Algeria, Tunisia, Israel, Morocco	Herbal tea from *A. herba*-*alba *has been used as analgesic, antibacterial, antispasmodic, and hemostatic agents in folk medicines	Camphor (17–33%), *α*-thujone (7–28%), and chrysanthenone (4–19%)	[9]

*Artemisia judaica* L.	Wormwood	Leaves	Egypt	Used for gastrointestinal disorders	Flavonoids with antioxidant activities.	[410]

*Callilepis laureola* DC.	Ox-eye daisy, Impila	Tuber	South Africa	Antioxidant and radical scavenging activities. Used to induce fertility, impotence, tapeworm infestations but induces hepatic and renal tubular necrosis.	Not identified	[188, 411, 412]

*Psiadia punctulata* (DC) Vatke	Mwendathigo	Leaf exudate	Kenya, East Africa	Used to treat colds, fevers and abdominals pains.	Flavones: 5,7-dihydroxy-2′,3′,4′,5′-tetramethoxyflavone, 5,4′-dihydroxy-7,2′,3′,5′-tetramethoxyflavone, 5,7,4′-trihydroxy-2′,3′,5′-trimethoxyflavone, 5-hydroxy-7,2′,3′,4′,5′-pentamethoxyflavone and 5,7,3′-trihydroxy-2′,4′,5′-trimethoxyflavone.	[359, 413]

*Vernonia kotschyana* Sch. Bip. ex Walp.	Buaye	Leaves, roots	Mali	Anti-inflammatory activity. Used to treat gastritis, gastro duodenal ulcers, as an aid to ameliorate digestion and as a wound healing remedy. Immunomodulating activities.	Not identified	[187, 414]

**Bignoniaceae**						

*Kigelia pinnata* DC.	Suasage tree, Cucumber tree	Root fruit	Egypt	Used as dressing for ulcers and used to treat rheumatism Anti-inflammatory activity	Naphthoquinones: kigelinone, isopinnatal, dehydro-alpha-lapachone, and lapachol and the phenylpropanoids: p-coumaric acid, ferulic acid (root), kigelinone and caffeic acid (fruits).	[415, 416]

*Tabebuia rosea* (Bertol.) DC.	Pink tecoma Pink trumpet tree	Leaves Stem-bark	Nigeria	Used to treat arthritis.	Tannins, flavonoids, alkaloids, quinones and traces of saponins	[107]

*Crescentia cujete* L.	Calabash Gourd tree	Leaves Stem-bark	Nigeria	Used as purgative and to treat coughs.	Tannins, flavonoids, alkaloids, quinones and traces of saponins	[107]

**Bombacaceae**						

*Bombax costatum* Pellegrin & Vuillet	Not signalized	Fruit	Niger	Antioxidant activity	Not identified	[11]

**Boraginaceae**						

*Heliotropium* *indicum *L.	Nonsikou	Leaves	Mali	Moderate antioxidant activity. Used for wound healing and for ocular infection.	Not identified	[417–419]

**Buddlejaceae**						

*Buddleja* *madagascariensis* Lam.	Butterfly-bush	Leaves	Egypt	Used to treat coughs, asthma, and bronchitis.	Flavonoids triglycosides: hesperetin and diosmetin 7-O (2′′,6′′- di-O-alpha-L-rhamnopyranosyl)-beta-D-glucopyranosides	[420]

**Caesalpiniaceae**						

*Cassia fistula* L.	Golden shower tree	Fruit	Mauritius	Laxative.	Phenolics and flavonoids	[368]

**Canellaceae**						

*Warburgia salutaris* (Bertol F.) Chiov.	Pepper-bark tree Isibaha	Bark	South Africa	Antioxidant and radical scavenging activities. Used to treat coughs, stomach ulcers, malaria, rheumatism, liver and venereal diseases	Not identified	[188]

*W. ugandensis *Sprague	Fever tree	Stem-bark Leaves	Kenya Ethiopia	Used to treat stomach ache, chest pains, malaria, toothache and coughs.	Flavonol glycoside Kaempferol, kaempferol 3-rhamnoside, kaempferol 3-Rhamnoside-7,4′-digalactoside and Quercetin: 3-Rhamnosyl(1→6[glucosyl(1→2)glucoside]-7-rhamnoside, kaempferide 3-O-beta-xylosyl (1→2)-beta-glucoside, kaempferol 3-O-alpha-rhamnoside-7,4′-di-O-beta-galactoside, kaempferol 3,7,4′-tri-O-beta-glucoside, kaempferol 3-rutinoside, myricetin, quercetin 3-rhamnoside, kaempferol 3-arabinoside, quercetin 3-glucoside, quercetin, kaempferol 3-rhamnoside-4′-galactoside, myricetin 3-galactoside and kaempferol 3-glucoside.	[421–424]

**Capparaceae**						

*Boscia senegalensis* (Pers.) Lam. ex Poiret	Senegal Boscia	Fruit hull Roots and leaf	Mali Niger	Antioxidant activity. Used to treat diarrhoea, cholera, tachycardia, pectoral pain.	Not identified	[12]

*Gynandropsis gynandra* Merr.	Not signalized	Leaves	Niger	Antioxidant activity	Not identified	[11]

**Celastraceae**						

*Salacia leptoclada* Tul.	Lemon rope	Root	South Africa	Antioxidant activity. Used as an aphrodisiac.	Not identified	[188]

**Chenopodiaceae**						

*Salsola somalensis *N.E.Br.	Dingetegna	Roots	Ethiopia	Used as taenicide.	Nine new isoflavones, 5,3′-dihydroxy-6,7,2′-trimethoxy isoflavone, 5,8,3′-trihydroxy-7,2′-dimethoxyisoflavone, 8,3′-dihydroxy-5,7,2′-trimethoxyisoflavone, 5,6,3′-trihydroxy-7,2′-dimethoxyisoflavone, 6,7,3′ -trihydroxy-5,2′-dimethoxyisoflavone, 5,8,3′-trihydroxy -2′-methoxy-6,7-methylenedioxyisoflavone, or 5,6,3′-trihydroxy-2′-methoxy-7,8-methylenedioxyisoflavone, 3′-hydroxy-5,6,7,2′-tetramethoxyisoflavone, 7,3′-dihydroxy -5,6,2′-trimethoxyisoflavone and 6,3′-dihydroxy-5,7,2′-trimethoxyisoflavone.	[425]

**Clusiaceae**						

*Psorospermum guineense* Hochr.	Karidjakouma	Leaves	Mali	Antioxidant activity. Used as diuretic and febrifuge.	Not identified	

**Combretaceae**						

*Pteleopsis suberosa* Engl. & Diels.	Girga	Stem-bark	Mali	Antioxidant properties. Used to treat gastric and duodenal ulcers.	Not identified	[329, 426]

**Dioscoreaceae**						

*Dioscorea dumetorum* Th.Dur.et Schinz	Cluster yam African bitter yam Trifoliate yam	Tubers	Nigeria Tropical West Africa	Antioxidant and hypolipidemic activities. Used to treat diabetes.	Not identified	[152, 153, 427]

**Ebenaceae**						

*Diospyros abyssinica* (Hiern) F. White	Giant diospyros	Leaves, roots Root-bark	Mali	Radical scavengers and lipoxygenase inhibitors.	Not identified	[357]

*Euclea divinorum *Hiern	Diamond-leaved euclea Magic guarri	Roots	Ethiopia	Used to treat venereal diseases, chest pains, pneumonia, internal body pains, stomach-ache and diarrhea. Chewed roots ease toothache.	Flavonoids	[428]

**Euphorbiaceae**						

*Acalypha hispida Burm. f.*	Chenille plant Red-hot cattail	Leaves Flowers	Nigeria	Used as anti-bacterial agent.	Gallic acid and Quercetin 3-O-rutinoside and kaempferol 3-O-rutinoside The main anthocyanin is the known cyanidind 3-*O*-(2-*O*-galloylgalactose, but a minor pigment (5%) is the new cyanidin Cy 3-*O-*(2-*O*-galloyl-6-*O-*rhamnosylgalactoside	[228, 429]

*A. wilkesiana* Müll. Arg.	Copper leaf	Leaves	Nigeria	Used to treat ailments of microbial origin	Gallic acid and Quercetin 3-O-rutinoside and kaempferol 3-O-rutinoside	[430]

*Croton gratissimus* Burch.	Lavender fever-berry	Bark	South Africa	Used as purgative for abdominal disorders, fever. The charred and powdered bark is used to treat bleeding gums	Flavonoids.	[188]

*Euphorbia hirta* L.	Kasandasanda Ufu idire	Whole plant Leaves	Ethiopia	Used to treat diarrhoea and asthma.	**Flavonoid**: quercitrin Flavonol: Euphorbianin (3-(6′′′-Acetylglucosyl) (1→3)galactoside)	[12, 431–433]

**Fabaceae**						

*Acacia caffra* (Thunb.) Wild.	Hook-thorn Cat-thorn	Bark	South Africa	Used to treat diarrhoea and as emetics.	Proanthocyanidins: oritin-(4alpha→5)-epioritin-4beta-ol, ent-epioritin-(4alpha→5)-epioritin-4beta-ol and epioritin-(4beta→5)-epioritin-4alpha-ol and ent-oritin-(4beta→5)-epioritin-4alpha-ol.	[434–436]

*A. galpinii* Burtt Davy.	Monkey-thorn	Bark	South Africa	Used to treat diarrhoea.	Proanthocyanidins: oritin-(4alpha→5)-epioritin-4beta-ol, ent-epioritin-(4alpha→5)-epioritin-4beta-ol and epioritin-(4beta→5)-epioritin-4alpha-ol and ent-oritin-(4beta→5)-epioritin-4alpha-ol.	[434, 435]

*Afzelia bella* Harms	Pretty Afzelia	Stem-bark	Ivory Coast	Used to treat skin diseases and cough.	An acylated dihydroflavonol glycoside identified as 2R,3R-trans-aromadendrin-7-O-beta-D-glucopyranoside-6′′-(4′′-hydroxy-2′′-m ethylene flavonoids:butanoate), along with five known flavonoids and the lignan glycoside (+)-isolariciresinol 9-O-xyloside.	[437]

*Bolusanthus speciosus* Harms	Tree Wisteria	Root Stem-bark	South Africa, Botswana, Mozambique, Zimbabwe, Zambia.	Used to treat abdominal pains, emetism and tuberculosis.	Three new flavonoids from the root: 5,7,4′-trihydroxy-6-[1-hydroxy-2-methylbuten-2-yl]isoflavone (isogancaonin C), 7,2′-dihydroxy-4′-methoxyisoflav-3-ene (bolusanthin III), 6,6′-dihydroxy-4′-methoxy-2-arylbenzofuran (bolusanthin IV) in addition to eight known derrone, medicarpan, genistein, wighteone, lupiwighteone, gancaonin C, 7-hydroxy-4′-methoxyisoflavone and 7,3′-dihydroxy-4′-methoxyisoflavone flavonoids 2*R*,3*R*-Aromadendrin 7-(6-[4-hydroxy-2-methylenebutanoyl]glucoside). Two new **i**soflavonoids from the combined ethyl acetate/methanolic extracts of the stem bark of Bolusanthus speciosus have been established as 4,7,2′-trihydroxy-4′methoxyisoflavanol (**1**) and 5,7,3′,4′-tetrahydroxy-5′-(2-epoxy-3-methylbutyl)isoflavanone (**2**). Five other known isoflavonoids, 5,7,3′-trihydroxy-4′-methoxy-5′-*γ*, *γ*-dimethylallyisoflavanone, 5,7,2′trihydroxy-4′-methoxy-6,5′-di(*γ*, *γ*-dimethyla)isoflavanone, 5,7,2′,4′-tetrahydroxy-8,5′-di(*γ*, *γ*-dimethylallyl)isoflavanone, 5,7,2′,4′-tetrahydroxy-8,3′-di(*γ*, *γ*-dimethylallyl)-isoflavanone, and derrone.	[67, 358, 438]

*Crotalaria lanceolata* E. Mey.	Lanceleaf rattlebox	Root	South Africa	Antioxidant activity. Used to treat coughs.	Not identified	[188]

*Derris trifoliata *Lour.	Common derris	Root-bark. Stem-bark. Seeds.	Kenya	Used for prevention of cancer. Entire plant is used as stimulant, antispasmodic. Bark is used as an alternative in rheumatism.	An isoflavonoid derivative, named 7a-O-methyldeguelol, a modified rotenoid with an open ring-C, representing a new sub-class of isoflavonoids (the sub-class is here named as rotenoloid). In addition, the known rotenoids, rotenone, deguelin and alpha-toxicarol. In addition, two unusual rotenoid derivatives, a rotenoloid (named 7a-O-methyl-12a-hydroxydeguelol) and a spirohomooxarotenoid (named spiro-13-homo-13-oxaelliptone). In addition a rare natural chromanone (6,7-dimethoxy-4-chromanone) and the known rotenoids rotenone, tephrosin and dehydrodeguelin were identified. Also one new rotenoid, 6-alpha,12-alpha-12a-hydroxyelliptone.	[438–441]

*Entada africana* Guill. & Perr.	Samanere	Leaves	Mali Niger	Antioxidant properties. Protective against carbon tetrachloride-induced liver damage. Used to treat fever and various respiratory diseases.	Not identified	[329, 357, 442, 443]

*Erythrina abyssinica* Lam.	Red hot poker tree	Stem bark Root bark	Kenya	Used to treat malaria.	New isoflav-3-ene [7,4′-dihydroxy-2′,5′-dimethoxyisoflav-3-ene] in addition to the known compounds erycristagallin, licoagrochalcone A, octacosyl ferulate and triacontyl 4-hydroxycinnamate were identified. A new chalcone, 2′,3,4,4′-tetrahydroxy-5-prenylchalcone (trivial name 5-prenylbutein) and a new flavanone, 4′,7-dihydroxy-3′-methoxy-5′-prenylflavanone (trivial name, 5-deoxyabyssinin II) along with known flavonoids	[444, 445]

*E. burttii* Baker f.	Not signalized	Stem-bark Root-bark	Kenya	Used as antifungal and antibacterial agent.	Two new flavanones: 5,7- dihydroxy-4′-methoxy-3′,5′-di-(3-methylbut-2-enyl)flavanone (trivial name, abyssinone V-4′-methyl ether) and 5,7-dihydroxy-4′-methoxy-3′-(3-hydroxy-3-methylbut-1-enyl)-5′-(3-methylbut-2-enyl)favanone (trivial name, burttinone). A new isoflavone, 5,2′,4′-trihydroxy-7-methoxy-6-(3-methylbut-2-enyl)isoflavone (trivial name, 7-O-methylluteone) and a new flavanone, 5,7-dihydroxy-4′-methoxy-3′-(3-methylbutadienyl)-5′-(3-methylbut-2-enyl)flavanone, 3 isoflavonoids (8-prenylluteone, 3-O-methylcalopocarpin and genistein) Three isoflav-3-enes, 7,4′-dihydroxy-2′-methoxy-6-(1′′,1′′-dimethylallyl)isoflav-3-ene (trivial name, burttinol-A), 4′-hydroxy-2′-methoxy-2′′,2′′-dimethylpyrano[5′′,6′′:8,7]isoflav-3-ene (trivial name, burttinol-B), 7,4′-dihydroxy-2′-methoxy-8-(3′′,3′′-dimethylallyl)isoflav-3-ene (trivial name, burttinol-C), and 2-arylbenzofuran, 6,4′-dihydroxy-2′-methoxy-5-(1′′,1′′-dimethylallyl)-2-arylbenzofuran (trivial name, burttinol-D).	[446–449]

*E. eriotricha* Harms.	Not signalized	Root-bark	Cameroon	Anti-microbial activity	A novel isoflavanone, named eriotrichin B, one new prenylated flavanone, named sigmoidin L, one flavanone (sigmoidin A), four isoflavones (scandenone, 6,8-diprenylgenistein), flemiphilippinin B and 8-prenyldaidzein	[450, 451]

*E. sacleuxii* Hua	Kinyarwanda	Bark	Kenya	Used to treat fever, malaria and leprosy.	Two new isoflavanones, (R)-5,7-dihydroxy-2′,4′,5′-trimethoxyisoflavanone (trivial name, (R)-2,3-dihydro-7-demethylrobustigenin) and (R)-5-hydroxy-2′,4′,5′-trimethoxy-2′′,2′′-dimethylpyrano[5′′,6′′:6,7]isoflavan one (trivial name, (R)-saclenone)	[452, 453]

*Millettia ferruginea* (Hochst.) Baker	Birbira Sotallo Sari	Bark	Ethiopia	Used for skin disorders.	O-Geranylated and O-prenylated flavonoids, C-prenylated isoflavones Geranylated and prenylated flavonoids	[199]

*M. dura *Dunn.	Runyankore Uumuyogoro	Stem-bark	Rwanda Uganda	Used for blood parasitism	Flavonoids: A new isoflavone (7,3′-dimethoxy-4′,5′-methylenedioxyisoflavone) and three known isoflavones [isoerythrinin A 4′-(3-methylbut-2-enyl) ether, isojamaicin and nordurlettone].	[454, 455]

*Ostryoderris stuhlmannii *(Taub.) Dunn ex Harms	Mnyinga	Leaves	Mali	Antioxidant activity. Used to treat painful menstruation, peritonitis, gastritis, colitis and gingivitis.	Not identified	[357]

*Piliostigma reticulatum *(DC.) Hochst	Kalga	Leaves Bark	Nigeria	High antioxidant activity. Used to treat wounds, bronchitis, malaria, sterility (leaves) and diarrhoea and dysentery (bark).	Not identified	[240]

*Sesbania pachycarpa* DC.	Not signalized	Leaves	Niger	Antioxidant activity	Not identified	[11]

*Tephrosia polyphylla* (Chiov.) J.B. Gillett	Hoary pea	Aerial part	Kenya		Flavonoids	[456]

*T. deflexa* Baker	Hoary pea	Aerial part	Senegal		Flavonoids: Rutin 1 – quercetine 3-O-a-L-rhamnopyrannosyl (1-6) glucopyrannose – and morin 2 – 3,5,7,2′,4′-pentahydroxyflavone.	[457]

*T. albifoliolis* A.Nongonierma & T.Sarr	Hoary pea	Aerial part	Senegal		Flavonoids: Rutin 1 – quercetine 3-O-a-L-rhamnopyrannosyl (1-6) glucopyrannose – and morin 2 – 3,5,7,2′,4′-pentahydroxyflavone.	[457]

*Taverniera abyssinica *A. Rich.	Dingetegna	Root	Ethiopia	Used to treat fever, discomfort and pain, stomach ache.	Four isoflavonoids	[290, 458, 459]

**Flacourtiaceae**						

*Flacourtia flavescens* Willd.	Not signalized	Leaves	Mali	Antioxidant activity.	Not identified	[357]

**Geraniaceae**						

*Pelargonium reniforme* Spreng.	Xhosa (Umckaloabo)	Root	Southern Africa	Used to treat liver disorders, laxative, purgative, cancer, and pulmonary disorders	Polyphenols: catechol (3′4′-dihydroxy) element in the B-ring, which possesses higher antioxidant activity than ascorbic acid.	[362, 460, 461]

**Gunneraceae**						

*Gunnera perpensa* L.	River pumpkin Ugobho	Root Leaves and stem.	South Africa	Decreased lucigenin enhanced chemiluminescence. Used to treat wounds and psoriasis.	Not identified	[21, 462]

**Irvingiaceae**						

*Irvingia gabonensis *(Aubry-Lecomte ex O'Rorke) Baill.	Bush mango Ono	Seeds	Nigeria Cameroon	Antioxidant activity. Used as laxative and for stomach and kidney pain. Shown to lower total cholesterol.	Not identified	[12, 313, 463]

**Lamiaceae**						

*Leonotis leonurus* (L.)R.Br.	Wild dagga	Leaves	South Africa	Anti-inflammatory properties. Used to treat headaches, dysentery, coughs and colds.	Not identified	[13]

*Salvia stenophylla *Burch. ex Benth.	Sage	Leaves	South Africa	Solvent extracts: antioxidant activity but poor anti-inflammatory properties. Essential oils: anti-inflammatory activity but poor anti-oxidant activity. Used against fever and digestive disorders.	Not identified	[360]

*S. repens *Burch. ex Benth.	Not signalized	Leaves	South Africa	Solvent extracts: antioxidant activity but poor anti-inflammatory properties. Essential oils: anti-inflammatory activity but poor anti-oxidant activity. Used for fevers and digestive disorders.	Not identified	[360]

*S. runcinata *L.f.	Not signalized	Leaves	South Africa	Solvent extracts: antioxidant activity but poor anti-inflammatory properties. Essential oils: anti-inflammatory activity but poor anti-oxidant activity. Used against fever and digestive disorders.	Not identified	[360]

**Loranthaceae **						

*Tapinanthus globiferus* Tiegh.	Not signalized	Leaves	Niger	Antioxidant activity	Not identified	[11]

**Malvacea**						

*Adansonia digitata *(L.)	English: baobab, Afrikaans: kremetart, Hausa: kuka, Sotho: seboi, Tswana: mowana, Tsonga: shimuwu, Venda: muvhuyu, Arabic: tabladi	Leaves, root, bark and fruits	All over Africa, but limited trees in Central Africa	Antioxidant, analgesic and anti-inflammatory properties of extracts	L-ascorbic acid	[36, 464]

**Mimosaceae**						

*Albizia lebbeck* (L.) Benth.	East Indian walnut, frywood, koko, lebbek, lebbek tree, rain tree, raom tree, silver raintree, siris rain tree, siris tree, soros-tree, woman's tongue.	Leaves and bark	Egypt	Used to treat asthma and skin disorders (bark) and eye diseases and dysentery (leaves)	Two new tri-O-glycoside flavonols: kaempferol and quercetin 3-O-alpha-rhamnopyranosyl(1→6)-beta-glucopyranosyl(1→6)-beta- galactopyranosides	[465]

**Moraceae**						

*Dorstenia angusticornis* Engl.	Not signalized	Twigs	Cameroon	Used for snakebite and to treat infection, rheumatism, headache, cough and stomach pain.	Two novel **diprenylated chalcones**: 3,5′-di-(2-hydroxy-3-methylbut-3-enyl)-4,2′,4′-trihydroxychalcone, 3, 4-(2,2-dimethylpyrano)-3′-(2-hydroxy-3-methylbut-3-enyl)-2′,4′-dihydroxych alcone and the known stipulin. 3-(2-Hydroxy-3-methylbut-3-enyl)-5′-(3,3-dimethylallyl)-4,2′,4′-trihydroxy chalcone and the known compounds: gancaonin Q, paratocarpins C, F, and lupeol.	[67, 278]

*D. dinklagei* Engl.	Not signalized	Twigs	Cameroon	Used for snakebite and to treat infection, rheumatism, headache, cough and stomach pain.	Three prenylated flavonoids, dinklagins A, B and C identified, respectively, as (dinklagin B): (+)-5,4′,5′′*ξ*-Trihydroxy-6′′,6′′-dimethyldihydropyranol[2′′,3′′:7,6]flavone. (dinklagin C): (+)-6-(2*ξ*-Hydroxy-3-methyl-3-butenyl)-5,7,4′-trihydroxyflavone (-)-6-(3,3-dimethylallyl)-7-hydroxy-6′′′, 6′′′-dimethylchromeno-(4′,3′,2′′′,3′′′)-flavanone, (+)-5,4′,5′′*ξ*-trihydroxy-6′′,6′′-dimethylchromano-(7,6,2′′,3′′)-flavone and (+)6-(2*ξ*-hydroxy-3-methyl-3-butenyl)-5,7,4′-trihydroxyflavone. 6-prenylapigenin, 4-hydroxylonchocarpin, stipulin and 5,4′-dihydroxy-6′′,6′′-dimethylchromano-(7,6,2′′,3′′)-flavone.	[67, 226]

*D. elliptica* Bur.	Not signalized	Twigs	Botswana	Used to treat eye infection.	Monoprenylated flavan	[466]

*D. Kameruniana*. Engl.	Not signalized	Leaves	Botswana	Used for snakebite and to treat infection, rheumatism, headache, cough and stomach pain.	Two novel favonoids: 6,7-(2,2-dimethylchromano)-5,4′-dihydroxyfavone and 3,4-,4′,5′-*bis*-(2,2-dimethylchromano)-2′-hydroxychalcone together with the known 6-(3-methylbut-2-enyl)apigenin and two chalcones (*E*)-1-[2,4-dihydroxy-3-[3-methylbut-2-enyl]phenyl]-3-[4-hydroxyphenyl]-prop-2-en-1-one and (*E*)1-[2,4-dihydroxy-5-[3-methylbut-2-enyl]phenyl]-3-[4-hydroxy-3-[3-methylbut-2-enyl]phenyl]-prop-2-en-1-one.	[467]

*D. prorepens* Engl.	Not signalized	Twigs	Botswana	Used for snakebite and to treat infection, rheumatism, headache, cough and stomach pain.	Digeranylated chalcone, 5,3′-(3,7-dimethyl-2,6-octadienyl)-3,4, 2′,4′-tetrahydroxychalcone. 4-Hydroxylonchocarpin Chalcone: 3,4,2′,4′-Tetrahydroxy-5,3′-digeranylchalcone	[67, 468]

*D. poinsettiifolia* Engl.	Not signalized	Twigs	Botswana	Used for snakebite and to treat infection, rheumatism, headache, cough and stomach pain.	Grenylated and prenylated flavonoids. In addition, the flavone 5,7,4-trihydroxy-8-prenylflavone (licoflavone C), the chalcones 4,2′,4′-trihydroxy-3′-prenylchalcone (isobavachalcone) and isobavachromene, the triterpene butyrospermol, and the carotenoid lutein.	[67, 206, 289]

*D. zenkeri* Engl.	Not signalized	Twigs	Botswana	Used for snakebite and to treat infection, rheumatism, headache, cough and stomach pain.	3′,4′-(3-hydroxy-2,2-dimethyldihydropyrano)-4,2′-dihydroxychalcone and a bichalcone. 4-Hydroxylonchocarpin. p-hydroxybenzaldehyde, dorsmanin A, 4,2′,4′-trihydroxychalcone and 4,2′,4′-trihydroxy-3′-prenylchalcone Chalcones: 4,2′,5′′-Trihydroxy-6′′,6′′-dimethyldihydropyranol[2′′,3′′:4′,3′]chalcone	[67, 468]

**Moringaceae**						

*Moringa oleifera* Lam.	Horse-radish tree Drumstick Moringo Zakalanda	Root	West Africa Zimbabwe	Anti-inflammatory activity. Used as aphrodisiac and to treat asthma, gout and rheumatism.	Not identified	[469]

*Myrtaceae *						

*Eucalyptus camaldulensis* Dehnh.	Not signalized	Leaves	Egypt	Antioxidant activity	Not identified	[470]

**Polygonaceae**						

*Polygonum senegalense* Meisn.	Fotsimbarin'akoholahy	Leaves	Madagascar		Flavonoids: quercetin, kaempferol and luteolin and their glycosides such as dihydrochalcone glucoside and quercetin glycosides.	[413, 471]

*Rumex abyssinicus* Jacq.	Mekmeko	Leaves	N. Africa - Ethiopia	Anti-inflammatory properties Used to treat itching, skin eczema and leprosy.	Flavonoids.	[337, 472]

*R. nervosus *Vahl.,	Alcgango Dengogo	Leaves	Ethiopia	Anti-inflammatory properties. Used to treat acne, wounds, eczema, typhus and as an ophthalmic antiseptic.	Not identified	[337]

**Rubiaceae**						

*Nauclea latifolia* Smith	Pin Cushion Tree Ìgíyàà	Leaves and root	Nigeria	Used as anthelmintic and to treat malaria, fever, stomachache and liver diseases.	Proanthocyanidins.	[12, 58, 473–475]

**Solanaceae**						

*Datura stramonium* L.	Thorn-apple rwiziringa	Seeds	South Africa	Antioxidant activity. Used to treat asthma, headaches and wounds.	Not identified	[188]

**Tiliaceae**						

*Grewia occidentalis* L.	Cross-berry Four-corner	Bark	South Africa	Antioxidant activity. Used to treat bladder ailments, wounds, impotence and sterility, and to help in childbirth.	Not identified	[188]

**Vahliaceae**						

*Vahlia capensis* (L.f) Thunb.	Vahlia of the Cape	Zimbabwe		Used to treat bacterial infections.	Kaempferol, quercetin, afzelin, astragalin, quercitrin, isoquercitrin, rutin, gallic acid, chiro-inositol, dulcitol, and a novel biflavonoid, VC-15B (vahlia biflavone)	[475]

**Vitaceae**						

*Cyphostemma natalitium* (Szyszl.) J.v. d. Merwe	Tick-berry bush	Root	South Africa	Anti-inflammatory and anti-microbial agents with significant inhibition of COX-1	Not identified	[374]

*Rhoicissus digitata* Gilg. & Brandt	Wilde patatat	Roots, stems and leaves	South Africa	At high concentrations possessed some prooxidative properties. Anti-inflammatory and anti-microbial agents with significant inhibition of COX-1. Used to facilitate delivery.	Not identified	[364, 374]

*R. rhomboidea* (E. Meyer ex Harvey) Planchon	Glossy forest grape	Roots, stems and leaves	South Africa Mozambique	Radical scavenging activity, inhibitory effect on xanthine oxidase activity, prevention of lipid peroxidation and damage to DNA and ability to chelate iron. Anti-inflammatory through inhibition of COX-1.	Not identified	[364, 374]

*R. tomentosa* (Lam.) Wild & R.B.Drum.	Wild grape Forest Grape, Monkey rope,	Roots, stems and leaves	South Africa	Antioxidant and anti-inflammatory activities. Anti-inflammatory through inhibition of COX-1. Used to facilitate delivery.	Not identified	[364, 374]

*R. tridentata* (L.f.) Wild & Drum.	Bitter grape Bushman's grape Isinwazi	Roots, stems and leaves	South Africa : Venda	Radical scavenging activity, inhibitory effect on xanthine oxidase activity, prevention of lipid peroxidation and damage to DNA and ability to chelate iron. Anti-inflammatory through inhibition of COX-1. Used to treat colds, infertility and stomach ailments.	Not identified	[364, 374, 476]
